# Chimeric SARS-CoV-2 vaccine vectors stably incorporating the functional glycoprotein of VSV

**DOI:** 10.1016/j.isci.2026.117521

**Published:** 2026-09-16

**Authors:** Enja Tatjana Kipfer, Jacob Schön, Mohamed Chami, Stefan Finke, Martin Beer, Donata Hoffmann, Fabian Otte, David Hauser

**Affiliations:** 1 Molecular Virology, Department of Biomedicine, University of Basel, 4009 Basel, Switzerland; 2 Institute of Diagnostic Virology, Friedrich-Loeffler-Institut, 17493 Greifswald-Riems, Germany; 3 BioEM Lab, Biozentrum, University of Basel, 4058 Basel, Switzerland; 4 Institute of Molecular Virology and Cell Biology, Friedrich-Loeffler-Institut, 17493 Greifswald-Riems, Germany

**Keywords:** SARS-CoV-2, vaccine platform, bivalent viral vector, pseudotyping, dual-immunogenicity, VSV-G, glycoprotein, chimeric

## Abstract

Respiratory viruses, such as SARS-CoV-2, remain a global medical challenge, highlighting the need for adaptable vaccine platforms that elicit broad immunity while allowing flexible antigen design. Building on our established envelope-deleted (ΔE) SARS-CoV-2 vaccine candidate, we engineered chimeric variants that stably express the glycoprotein of vesicular stomatitis virus (VSV-G) as a model antigen, with or without co-expression of the native SARS-CoV-2 spike protein. Both constructs were genetically stable and displayed functional VSV-G, as shown by expanded, ACE2-independent tropism. Cryo-electron microscopy confirmed the presence of heterologous glycoproteins on virions. *In vivo*, both chimeric viruses exhibited an excellent safety profile and induced antigen-specific humoral responses against the encoded glycoproteins in Syrian hamsters and K18-hACE2 mice. Together, these findings demonstrate stable genetic incorporation and display of a functional heterologous glycoprotein into a SARS-CoV-2 virion, supporting use of the ΔE SARS-CoV-2 backbone as a potential platform for viral vector engineering and heterologous antigen delivery.

## Highlights

• ΔE SARS-CoV-2 vaccine viruses stably display a heterologous glycoprotein
• SARS-CoV-2/VSV chimeric virions exhibit expanded, ACE2-independent cell tropism
• Chimeric constructs are safe in two rodent models and induce antibody responses
• ΔE SARS-CoV-2 backbone offers a versatile platform for vaccine vector engineering

## Graphical abstract

**Figure:**
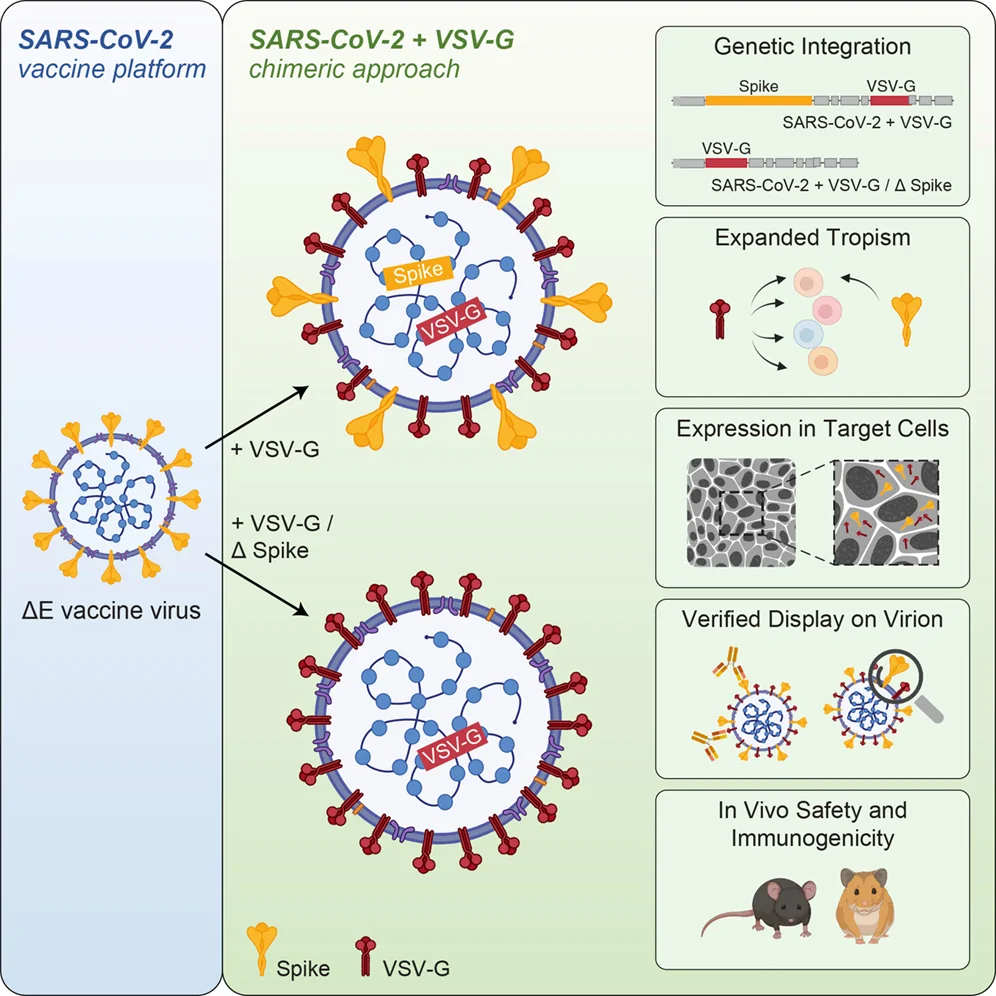

## Teaser

Immunology; Virology

## Introduction

The rapid global response to SARS-CoV-2, the causative agent of COVID-19,^1^ particularly during the early phase of the pandemic, highlighted the importance of pre-existing viral platform technologies and global scientific collaboration. Vaccine development began within months of the virus’s emergence, facilitated by viral vector systems originally developed for other pathogens and readily adaptable for SARS-CoV-2. This response demonstrated how diversified, well-characterized viral platforms, combined with international research networks, can form the backbone of an effective, rapid pandemic response.

Among the first vaccines deployed were mRNA-based formulations, which benefited from extensive prior development activities, originally designed to address topics in the cancer field, and the capacity for rapid antigen redesign, enabling accelerated clinical approval.^2^^,^^3^ Soon thereafter, more classical adenoviral-vector vaccines followed,^4^ as well as inactivated whole-virus vaccines,^5^ and protein-subunit^6^ or nanoparticle-based formulations.^7^ Together, this first wave of immunization strategies profoundly reduced severe disease and mortality, and contributed to the global control of COVID-19.^8^ However, the continuous antigenic evolution of the SARS-CoV-2 spike protein (S) has since generated viral immune-escape variants,^9^ and comparative analyses of mRNA-vaccinated and naturally infected individuals have revealed differences in the breadth of neutralizing antibody responses, highlighting limitations of spike-focused vaccines.^10^^,^^11^

In response, next-generation vaccine strategies increasingly emphasize whole-virus approaches that better mimic natural infection, including live-attenuated (LAV) and replication-restricted viruses. These platforms allow presentation of the full repertoire of viral antigens—not only spike, but also other structural and accessory proteins—potentially eliciting a broader and more durable humoral and cellular immune response.^10^^,^^12^^,^^13^ Indeed, multiple B and T cell epitopes have been identified for SARS-CoV-2, including membrane (M), nucleocapsid (N), and various accessory proteins involved in replication and immune modulation.^14^^,^^15^ These antigens are less susceptible to antigenic drift, a property that may be crucial for addressing recurrent outbreaks driven by emerging variants. Notably, several T cell epitopes are conserved among coronaviruses in general, which suggests the potential for cross-reactivity and durable immunity.^16^

SARS-CoV and SARS-CoV-2, with their large, 29 kb positive-sense RNA genome, have been explored as viral vector backbones capable of delivering foreign genetic sequences, as demonstrated by numerous reporter-engineered viruses.^17^^,^^18^^,^^19^^,^^20^^,^^21^^,^^22^^,^^23^^,^^24^ Their suitability as a vector is supported by high replication titers *in vitro*^25^ and the presence of a proofreading RNA polymerase, enhancing genetic stability.^26^ Recent work has demonstrated that SARS-CoV-2 can stably incorporate and express heterologous antigens, such as a prefusion-stabilized fusion (F) glycoprotein of respiratory syncytial virus (RSV), designed for secretion rather than virion incorporation, eliciting dual immune responses *in vivo* and supporting the use of SARS-CoV-2 for bivalent immunization strategies.^23^

Bivalent vaccine approaches may offer advantages as SARS-CoV-2 transitioned into an endemic, seasonal pathogen, co-circulating with other viruses. Such strategies could reduce the number of required applications and potentially limit co-infections that would lead to more severe clinical outcomes.

Spike-mediated viral entry depends on the angiotensin-converting enzyme 2 (ACE2), which is predominantly expressed in the respiratory tract and only minimally in skeletal muscle.^27^ This receptor dependency can limit the efficiency of intramuscular vaccination approaches. Incorporation or co-expression of heterologous viral glycoproteins with broader tropism, such as the vesicular stomatitis virus glycoprotein (VSV-G), can enable ACE2-independent entry and enhance cellular uptake and immunogenicity.^28^^,^^29^^,^^30^ This principle was exemplified by Merck’s chimeric live recombinant rVSVΔG-Spike vaccine candidate V590, which showed promising preclinical results in rodents but elicited limited immunogenicity following intramuscular administration in a phase 1 clinical trial, leading to its discontinuation.^31^^,^^32^^,^^33^ Subsequent studies in non-human primates demonstrated that co-expression of alternative glycoproteins, such as the Ebola virus glycoprotein, could restore immunogenicity upon intramuscular delivery, highlighting the critical role of viral tropism in vaccine efficacy.^34^

As viral tropism engineering for live SARS-CoV-2 will enable broader target delivery, thereby opening new application routes, such as intramuscular administration, and expanding study possibilities, we investigated whether live SARS-CoV-2-based vaccine vectors can stably incorporate a functional heterologous glycoprotein. We built on our recently developed *E*-gene deleted (ΔE) SARS-CoV-2 platform,^35^^,^^36^ a replication-competent but propagation-restricted vaccine system that can be grown to high titers in E protein-complementing cells yet remains limited to a single round of replication *in vivo*, providing excellent safety and transmission-blocking immunity *in vivo*.

Here, we genetically introduced the VSV glycoprotein, alone or in combination with the native spike, into the ΔE backbone to evaluate whether chimeric SARS-CoV-2 virions can incorporate and present a functional heterologous glycoprotein. We assessed genetic stability, producibility, and glycoprotein functionality *in vitro*, and characterized safety and immunogenicity in Syrian hamsters and K18-hACE2 mice.

This work aims to add to the growing portfolio of modular viral vaccine platforms by establishing a proof-of-concept for chimeric SARS-CoV-2 whole-virus vaccine vectors.

## Results

### Generation of infectious ΔE SARS-CoV-2 vectors expressing the *VSV-G* gene

The chimeric SARS-CoV-2-vectored viruses of this study were all based on a previously developed *E* gene-deleted (ΔE) vaccine backbone, which showed favorable safety and protection against challenge *in vivo*.^35^ In addition to *E*, *open reading frame (ORF) 6* and *ORF7a* were deleted, and a premature stop codon at amino acid position 213 in *ORF3a* was introduced to ensure genetic stability (ORF3a^213^^∗^). For simplicity, this ΔE ΔORF6/ΔORF7a ORF3a^213^^∗^ backbone is being termed here “ΔE”.

**Figure 1:**
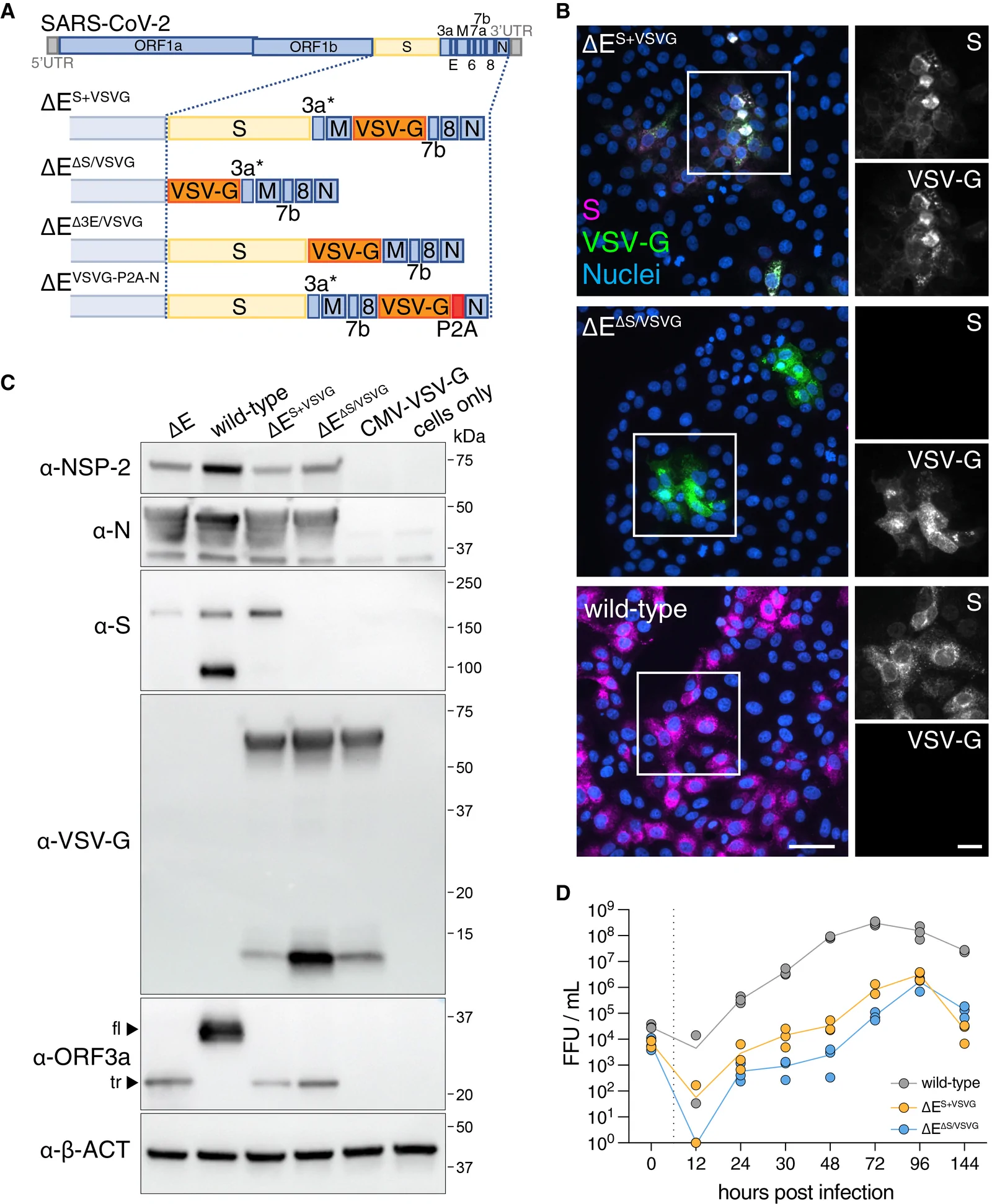
Characterization of chimeric SARS-CoV-2 (A) Schematic SARS-CoV-2 wild-type genome and its chimeric derivatives. VSV-glycoprotein gene (*VSV-G*, orange), *P2A* (red), structural genes, and *ORFs* are labeled accordingly. (B) Immunofluorescence stainings of SARS-CoV-2 S protein (magenta) and VSV-G (green) in VeroTMP cells 24 h post infection (hpi) with ΔE^S+VSVG^, ΔE^ΔS/VSVG^, and wild-type SARS-CoV-2 control. Nuclei are in blue. Scale bars represent 50 *μ*m in overview and 20 *μ*m in ROI images. (C) Representative immunoblot images of viral protein expression 24 hpi in VeroTMP cell lysates from infections with ΔE^S+VSVG^, ΔE^ΔS/VSVG^, wild-type SARS-CoV-2, and the ΔE vaccine virus or from VSV-G-transfected (positive control) and uninfected VeroTMP cells (negative control). β-actin (β-ACT) was used as a cellular loading control. Viral infection was confirmed by detection of nonstructural protein 2 (NSP2) and N. Expression of S, VSV-G, full-length (fl) ORF3a, and the truncated (tr) ORF3a^213^^∗^ variant was analyzed. Uncropped blots are shown in Figure S3. (D) Replication kinetics of ΔE^S+VSVG^ and ΔE^ΔS/VSVG^ compared with wild-type SARS-CoV-2. Vero E2T cells were infected in technical triplicate (*n* = 3) at a multiplicity of infection (MOI) of 0.01. Supernatants were collected at 12, 24, 30, 48, 72 (3 days), 96 (4 days), and 144 (6 days) hpi and quantified by focus forming assay (FFA). Cell monolayers were washed at 3 hpi to remove input virus (indicated by dashed line). Connecting lines show means at individual time points. See also Figures S1 and S3.

The *VSV-G* (Indiana strain) gene was inserted into the genetic background of the ΔE vaccine virus and rescued using the previously described CLEVER method.^37^ Four different viral constructs were generated with the foreign gene in various genomic loci (Figure 1A): (1) within the *ORF6* and *ORF7a* region, replacing *ORF6* and *ORF7a* (ΔE^S+VSV^^G^); (2) at the spike locus, replacing the *S* gene (ΔE^ΔS/VSV^^G^); (3) within the *E* and *ORF3a* region, replacing both the *E* gene and *ORF3a* (ΔE^ΔE3a/VSV^^G^); and (4) upstream of the *N* gene, with the *VSV-G* coding sequence followed by a P2A self-cleaving peptide and the *N* coding sequence (ΔE^VSV^^G-P2A^^-^^N^). Each of these resulted in a SARS-CoV-2-derived virus harboring either the two glycoproteins SARS-CoV-2 spike and VSV-G (1, 3, and 4) or only VSV-G (2).

All four constructs yielding VSV-G were infectious in Vero E6 cells stably expressing TMPRSS2 (VeroTMP), as demonstrated by immunofluorescence staining (Figure S1A). Sequencing of low-passage virus populations confirmed the successful integration of the heterologous glycoprotein gene in all four constructs (Table S1).

Robust virus production was achieved for constructs ΔE^S+VSVG^ and ΔE^ΔS/VSVG^ during serial passages in Vero E6 cells with stable E expression (Vero E2T), as indicated by pronounced cytopathic effect (CPE) and high virus titers. The viruses ΔE^VSV^^G-P2A^^-^^N^ and ΔE^ΔE3a/VSV^^G^ replicated only to very low titers, with few infectious particles produced (Figure S2A). Due to this limited replication capacity, subsequent analyses focused on high-titer constructs ΔE^S+VSVG^ and ΔE^ΔS/VSVG^, which maintained stable integration of the heterologous sequences, as well as the *S* gene in ΔE^S+VSVG^, for more than 10 passages (Table S1).

Targeted sequencing of the *S* gene and the downstream *ORFs* confirmed its stability with only a few mutations (Table S1). In ΔE^S+VSVG^, adaptive mutations emerged in the cytoplasmic tail of spike, including a single-nucleotide deletion (nt25353, codon 1270). Interestingly, an independently generated viral stock acquired a distinct four-nucleotide deletion (nt25354-25357), both deletions causing a frameshift that extended the spike C terminus by 21 amino acids. Because the altered cytoplasmic tail terminates with the amino acid sequence “FEAR”, these variants were designated FEAR^∗^. Analysis of early passage populations indicated that these mutations arose during extended culture and occurred independently in multiple replicates, with each mutation being genetically distinct, suggesting adaptive selection. Two replicates acquired an altered, prolonged cytoplasmic tail that did not result in FEAR^∗^. Viral populations lacking FEAR^∗^ mutations exhibited reduced viral titers (Table S2).

Complementary next-generation sequencing of the genomic backbone showed only a few minor mutations, summarized in Table S3. Mutations emerging recurrently across multiple populations are highlighted, with mutations still detectable in high-passage stocks considered relevant, such as F3677C in *ORF1ab* and G147R in *M* (ΔE^ΔS/VSVG^).

Overall, we show the successful rescue and infectivity of four genetically distinct chimeric viruses expressing the heterologous glycoprotein VSV-G. Furthermore, ΔE^S+VSVG^ and ΔE^ΔS/VSVG^ showed efficient replication and stable integration of the heterologous gene within the SARS-CoV-2 genome over several *in vitro* passages.

### Cellular expression and replication characteristics of chimeric SARS-CoV-2 constructs

Expression of the ectopic protein in target cells was examined by immunofluorescence staining (Figure 1B). VeroTMP cells were infected with ΔE^S+VSVG^ and ΔE^ΔS/VSVG^, and protein expression was compared to wild-type SARS-CoV-2 infections. According to the genetic structure, construct ΔE^S+VSVG^ expressed both spike and VSV-G, whereas ΔE^ΔS/VSVG^ expressed only VSV-G but not spike (Figure 1B). Additional viral protein expression, including N, ORF3a, and ORF8, as well as the absence of ORF6 and ORF7a, was consistent with the engineered constructs (Figures S1B–S1E). Immunoblot analysis of total cellular protein extracts further confirmed expression of VSV-G and spike proteins, as well as the expected truncated ORF3a resulting from the introduced stop codon in the ΔE vaccine viruses (Figures 1C and S3).

Replication kinetics of chimeric constructs were compared with wild-type SARS-CoV-2 on E-transcomplementing Vero E2T cells (Figure 1D). Supernatants collected over six days showed that both constructs displayed comparable growth kinetics, reaching peak titers of 3.15 × 10^6^ focus-forming units (FFU)/mL (ΔE^S+VSVG^) and 1.57 × 10^6^ FFU/mL (ΔE^ΔS/VSVG^) at 96 h post-infection (hpi). In contrast, wild-type SARS-CoV-2 achieved a peak titer of 2.97 × 10^8^ FFU/mL already at 72 hpi.

For subsequent studies, optimized culture conditions further increased titers to 1.5 × 10^7^ FFU/mL for ΔE^S+VSVG^ and 2.1 × 10^6^ FFU/mL for ΔE^ΔS/VSVG^ (Figures S2B and S2C), comparable to the parental ΔE vaccine virus (10^7^ FFU/mL). These findings indicate that, although the ΔE vaccine virus reaches slightly lower titers on producer cells *in vitro*, neither the introduced nucleotides nor the ectopic glycoprotein have a significant effect on viral fitness.

A strong attenuation and propagation incompetence of the chimeric E-deficient vaccine viruses was proved on non-transcomplementing human alveolar cells expressing ACE2 and TMPRSS2 (A549 AT), where only wild-type SARS-CoV-2 replicated to high titers, and both chimeric viruses remained below the detection limit of 10^3^ FFU/mL even after four passages (Figure S2D). No reversion of the *E* gene was observed after deep sequencing of high-passage virus populations (Figure S2E).

### VSV-G is incorporated in SARS-CoV-2 virions and displayed as a functional receptor

**Figure 2:**
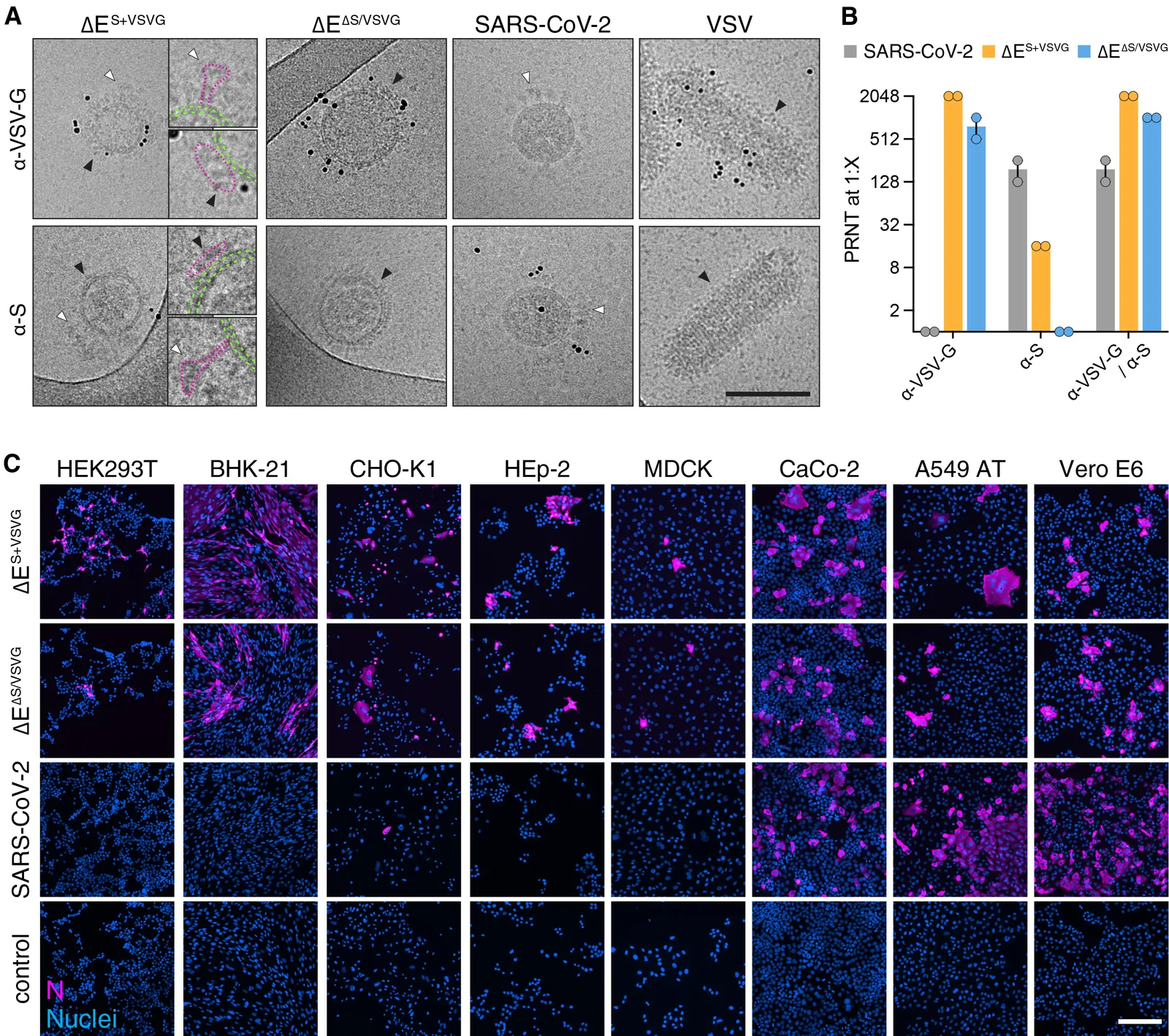
Structural and functional characterization of chimeric viruses (A) Immunogold cryo-EM of ΔE^S+VSVG^, ΔE^ΔS/VSVG^, wild-type SARS-CoV-2 and VSV incubated with α-VSV-G and α-spike, detected by gold-conjugated secondary antibody (6 nm). Representative images from *n* = 3 experiments are shown; additional replica and uncropped overview pictures are shown in Figure S4. (B) Neutralizing capacity of α-spike and α-VSV-G on ΔE^S+VSVG^ and ΔE^ΔS/VSVG^ compared to wild-type SARS-CoV-2. The viruses were incubated with the same amount of a serially diluted neutralizing antibody, and neutralizing capacity was estimated by comparing plaque numbers to control infections (2 technical replicates per condition; *n* = 1). Antibody dilutions with 90% neutralization/plaque number reduction are shown. Means are plotted, with bars indicating SEM. Original 96-well plates are shown in Figure S5A. (C) Cell tropism of ΔE^S+VSVG^ and ΔE^ΔS/VSVG^ compared to wild-type SARS-CoV-2. HEK293T, BHK-21, CHO-K1, HEp-2, MDCK, CaCo-2, A549 AT, and Vero E6 cells were seeded on coverslips, infected with ΔE^S+VSVG^, ΔE^ΔS/VSVG^, wild-type, or mock control. Cells were fixed 24 hpi and subsequently stained with α-N to visualize infection events (magenta). Nuclei are in blue. Representative images from *n* = 2 independent experiments are shown. Scale bars represent 100 nm (A) and 200 *μ*m (C). See also Figures S4, S5, and S6.

To gain structural and functional insights into VSV-G incorporation into virions, we performed immunogold labeling followed by cryo-electron microscopy (cryo-EM), antibody-mediated neutralization assays, and cell tropism analysis (Figures 2, S4, and S5).

Cryo-EM confirmed characteristic corona-shaped virions for wild-type SARS-CoV-2 viruses, while VSV virions appeared bullet-shaped with a smaller and more homogeneous display of VSV-G (Figures 2A and S4). ΔE^ΔS/VSVG^ virions displayed a uniform circular, SARS-CoV-2-like shape with smaller glycoproteins resembling VSV-G, while ΔE^S+VSVG^ virions displayed a heterogeneous surface structure, containing regions resembling VSV-G as well as areas with larger, spike-like structures comparable to S of SARS-CoV-2 (Figure 2A).

Immunogold labeling with α-VSV-G antibodies demonstrated labeling of wild-type VSV and of both chimeric virus preparations, indicating VSV-G incorporation into their envelopes. α-S labeling of the ΔE^S+VSVG^ virions and SARS-CoV-2 wild-type controls revealed only sparse signals, compatible with the lower binding affinity of α-S. Control stains had no gold particle binding, confirming the high specificity of the antibody labeling. SARS-CoV-2 particles typically measure 80–100 nm in diameter; however, the chimeric virions exhibited greater size variability, suggesting structural heterogeneity in the viral envelope.

To assess the structural integrity of the incorporated glycoproteins, engineered viruses were incubated with neutralizing antibodies targeting spike, VSV-G, or both, and plaque formation was analyzed (Figures 2B and S5A). α-VSV-G antibodies efficiently neutralized both chimeric viruses, confirming functional expression of VSV-G on the virion surface. α-Spike antibodies inhibited wild-type SARS-CoV-2, and to a lesser extent, the ΔE^S+VSVG^ variant, while showing no effect on the VSV-G-only construct ΔE^ΔS/VSVG^. Combining both antibodies inhibited the infection of all viruses. Analysis of infected foci following ΔE^S+VSVG^ infection further confirmed the dominant effect of VSV-G targeting antibodies, as neutralization by α-VSV-G alone was comparable to the combined antibody treatment (Figure S5B).

Viral glycoproteins mediate cell attachment and entry through interactions with specific host cell receptors, thereby determining the viral tropism. The incorporation of an ectopic VSV glycoprotein into heterologous viral particles would therefore be expected to expand tropism toward that of the introduced glycoprotein.

To test potential modified cell tropism by VSV-G expression, a diverse panel of mammalian cell lines was infected with ΔE^S+VSVG^, ΔE^ΔS/VSVG^, or wild-type SARS-CoV-2, including HEK293T, BHK-21, CHO-K1, HEp-2, and MDCK cells, as well as CaCo-2, A549 AT, and Vero E6 cells (Figure 2C). The latter three cell lines are known to be permissive for SARS-CoV-2. Infectivity, determined by detection of intracellular N, showed that wild-type SARS-CoV-2 infected CaCo-2, A549 AT, and Vero E6, and to a lesser extent, CHO-K1 cells. In contrast, both chimeric viruses infected all tested cell lines, similar to wild-type VSV infections (Figure S6). This is in line with the wider host range of VSV, demonstrating functional incorporation of VSV-G into the viral envelope and the corresponding tropism expansion.^38^

### Chimeric constructs are safe and immunogenic *in vivo*

**Figure 3:**
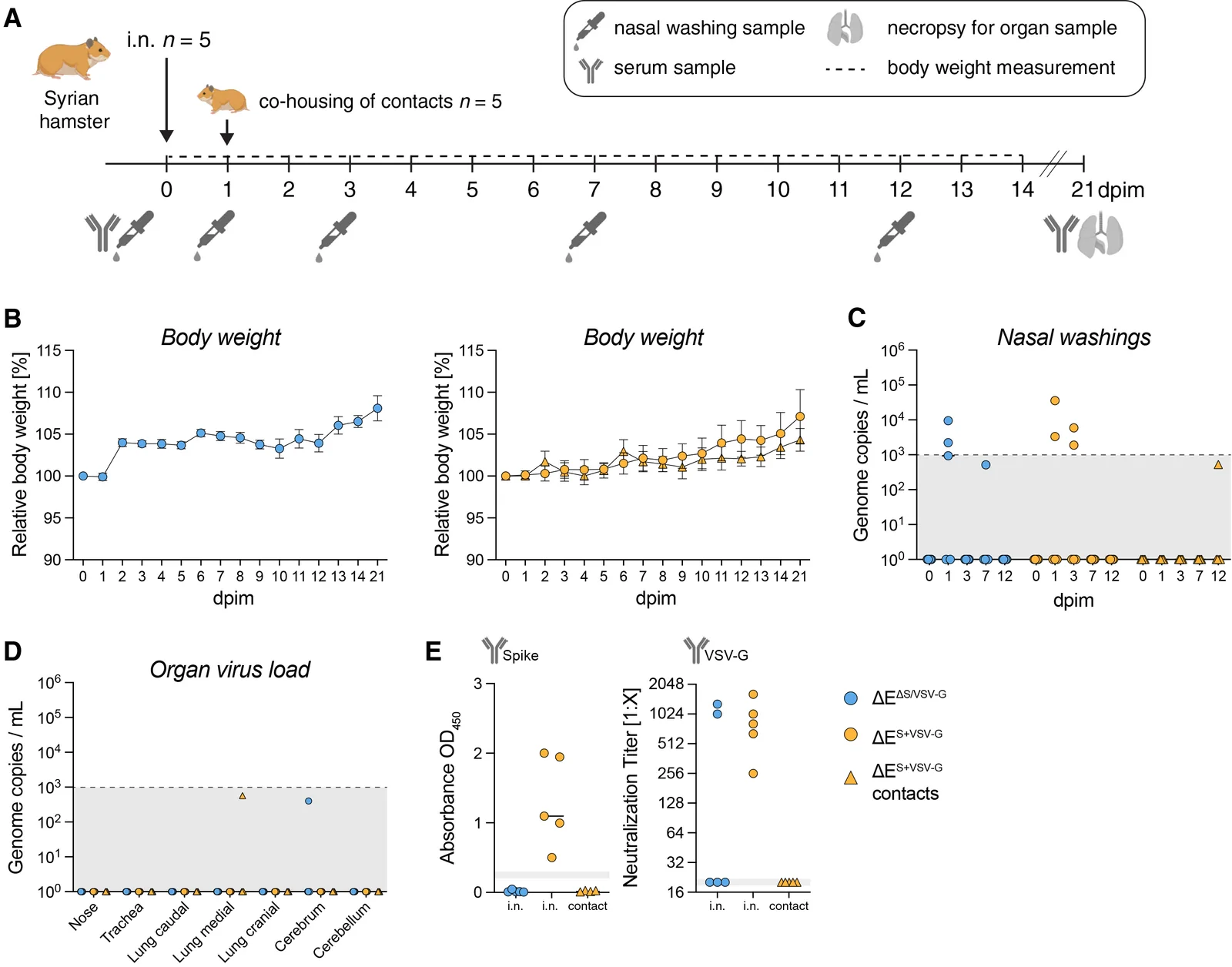
*In vivo* safety and immunogenicity in hamsters (A) Syrian hamsters (*n* = 5 per group) were intranasally (i.n.) or intramuscularly (i.m.) immunized with ΔE^S+VSVG^ or ΔE^ΔS/VSVG^, respectively; contact animals were added 1 day post immunization (dpim) (*n* = 5). Body weight was monitored, and nasal washings, blood samples, and organ samples were taken at the indicated time points. (B) Relative body weight after immunization. Means are plotted, with bars indicating SEM. (C) Quantification of SARS-CoV-2 genome copies (*ORF1b*) in nasal washings measured by RT-qPCR. (D) SARS-CoV-2 genome copies (*ORF1b*) in the nose, lung (caudal, medial, and cranial), cerebrum, and cerebellum at 21 dpim, measured by RT-qPCR. (E) Humoral antibody induction at 21 dpim. Left: spike RBD-specific IgG measured by ELISA (OD_450_); individual values and median are shown. Right: neutralization titers against wild-type VSV Indiana measured by live virus neutralization assay; individual values are shown. Gray area indicates values below the limit of detection (1,000 genome copies/mL) in (C) and (D), or below the seronegative threshold (serum dilution <1:20) in (E). See also Figures S2 and S7.

**Figure 4:**
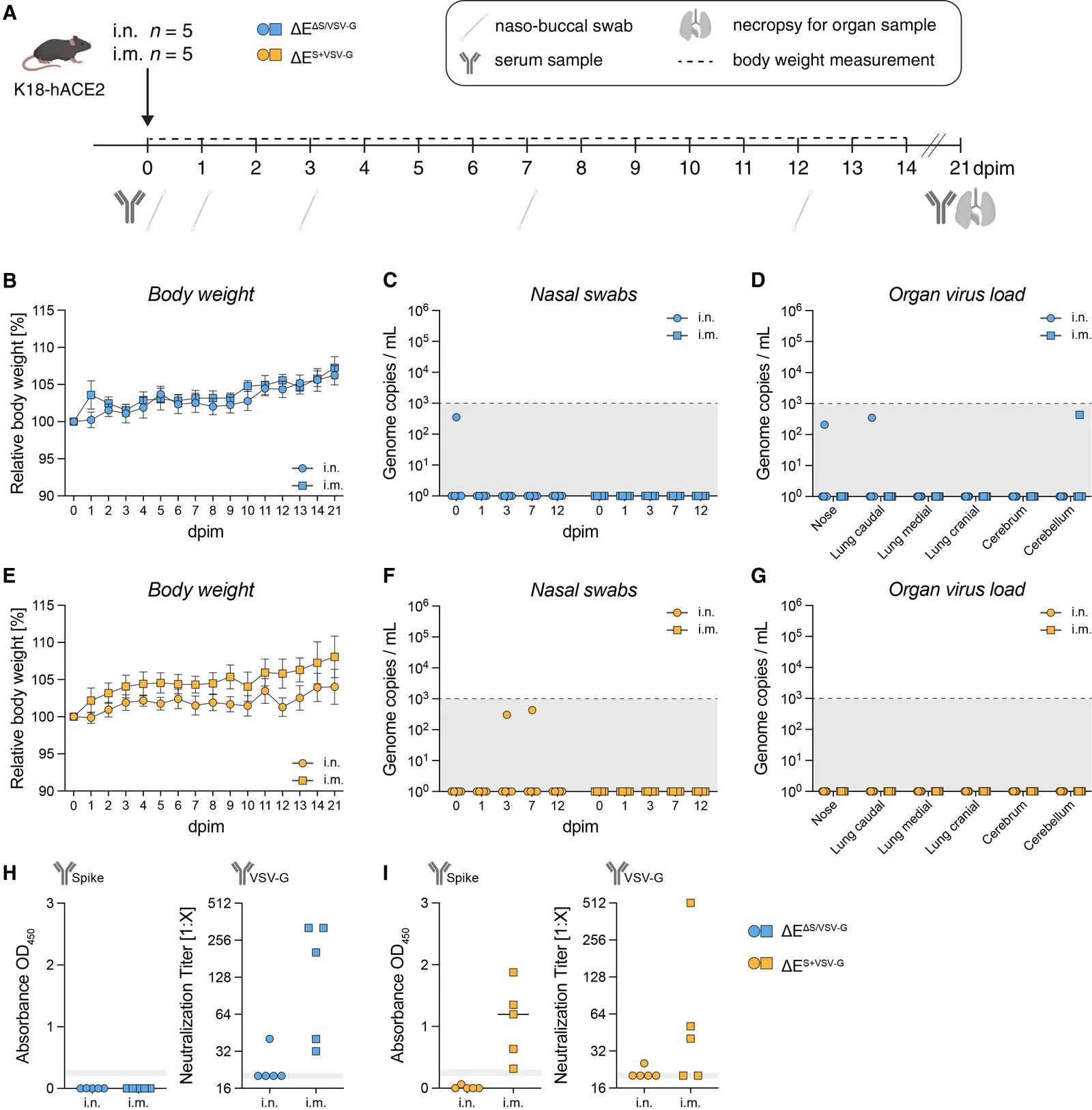
*In vivo* safety and immunogenicity in mice (A) K18-hACE2 mice (*n* = 5 per group) were intranasally (i.n.) or intramuscularly (i.m.) immunized with ΔE^S+VSVG^ or ΔE^ΔS/VSVG^, respectively. Body weight was monitored, and naso-buccal swabs, blood samples, and organ samples were taken at the indicated time points. (B and E) Relative body weight after immunization. Means are plotted, with bars indicating SEM. (C and F) Quantification of SARS-CoV-2 genome copies (*ORF1b*) in naso-buccal swabs measured by RT-qPCR. (D and G) SARS-CoV-2 genome copies (*ORF1b*) in the nose, lung (caudal, medial, and cranial), cerebrum, and cerebellum at 21 dpim, measured by RT-qPCR. (H and I) Humoral antibody induction at 21 dpim. Left: spike RBD-specific IgG measured by ELISA (OD_450_); individual values and median are shown. Right: neutralization titers against wild-type VSV Indiana measured by live virus neutralization assay; individual values are shown. Gray area indicates values below the limit of detection (1,000 genome copies/mL) in (C), (D), (F), and (G), or below the seronegative threshold (serum dilution <1:20) in (H) and (I). See also Figures S2 and S7.

The safety and immunogenicity of ΔE^S+VSVG^ and ΔE^ΔS/VSVG^ viruses were assessed in Syrian hamsters and K18-hACE2 transgenic mice (Figures 3 and 4). Syrian hamsters are naturally susceptible to SARS-CoV-2 infection and represent a sensitive transmission model.

Hamsters were inoculated intranasally (i.n.) with 5.25 × 10^4^ infectious units of ΔE^S+VSVG^ or ΔE^ΔS/VSVG^ (*n* = 5 per group). Naive contact animals (*n* = 5) were co-housed with ΔE^S+VSVG^-inoculated animals 1 day post immunization (dpim) (Figure 3A).

Body weight was monitored, and viral genome levels were quantified in nasal washings at defined time points. Organ and serum samples were analyzed at 21 dpim for viral genome copies and antibody responses, respectively. Both chimeric viruses were well tolerated, with no body weight loss in either the vaccinated or contact groups (Figure 3B).

Viral genome copies in the nose declined rapidly. For ΔE^ΔS/VSVG^, only 2/5 animals exhibited viral RNA above the detection limit at 1 dpim, with no quantifiable replication afterward (Figure 3C). For ΔE^S+VSVG^, 2 of 5 animals showed higher genome copy numbers at 1 and 3 dpim. Yet none of the vaccinated animals showed evidence of productive viral replication, as no viral RNA was detected in any group from 7 dpim onward.

No viral RNA was detected above the assay’s detection limit in contact animals. Furthermore, viral RNA levels remained below quantifiable limits in all organ samples at 21 dpim (Figure 3D).

Spike reactivity was measured by RBD-specific ELISA, and VSV-G-directed antibodies were evaluated by live virus neutralization assay (Figure 3E). All hamsters inoculated with ΔE^S+VSVG^ developed SARS-CoV-2 S-specific antibodies and had high neutralizing titers against wild-type VSV Indiana by 21 dpim. In contrast, no S-specific antibodies were detected in animals vaccinated with the S-deficient ΔE^ΔS/VSVG^, and only 2 out of 5 animals showed α-VSV titers (Figure 3E). All contact animals stayed seronegative in both assays, supporting the conclusion that no vaccine virus transmission occurred (Figure 3E).

To confirm safety and to evaluate additional administration routes, K18-hACE2 mice were vaccinated either i.n. or intramuscularly (i.m.) with equivalent doses of both constructs (5.25 × 10^4^ infectious units; *n* = 5 per group) and assessed for body weight, viral replication, and organ viral load (Figure 4A). As in the hamster model, no effect on body weight, no viral shedding, and no detection of viral RNA in organs could be measured for both chimeric viruses (Figures 4B–4G), confirming the excellent safety profile of ΔE-based vaccine viruses.

As in the hamster model, spike-lacking chimeric viruses did not induce antibodies against spike, regardless of the administration route, and only 1/5 animals produced responses against VSV when applied intranasally (Figure 4H). Contrary, intramuscular application of ΔE^ΔS/VSVG^ induced robust neutralization against wild-type VSV (Figure 4H).

For ΔE^S+VSVG^, intramuscular vaccination induced robust S-specific antibody responses in all animals, and VSV-neutralization in 3/5 animals (Figure 4I). When applied intranasally, only 1/5 animals developed a slightly elevated neutralizing titer against VSV (Figure 4I). Highly sensitive immunofluorescence assays recapitulated VSV neutralization results for both chimeric viruses (Figures S7A and S7B). Neutralization assay with live SARS-CoV-2 was performed on 21 dpim and did not detect neutralizing antibodies over detection limits (Figures S7C and S7D).

Together, these *in vivo* experiments demonstrate the excellent safety of ΔE-based vectors, with restricted replication and lack of transmission. In addition, chimeric SARS-CoV-2 vectors are capable of inducing antigen-specific immune responses. Thus, the modularity of the platform was further investigated by inserting a heterologous *Mycobacterium tuberculosis* antigen (esxA) into the ΔE backbone. Sequencing analysis confirmed stable integration across multiple passages, and mass spectrometry verified the expression of the encoded protein (Figure S8 and Table S4). These findings support the concept of the ΔE SARS-CoV-2 system as a genetically stable and versatile platform for the development of multivalent viral vectors.

## Discussion

This study describes the generation and characterization of SARS-CoV-2 vaccine viruses stably incorporating the heterologous VSV-G glycoprotein. Whereas pseudotyping of SARS-CoV-2 virus-like particles^39^ or replicons^40^ has been demonstrated previously, this is, to our knowledge, the first study describing the genetically stable incorporation of a functional foreign glycoprotein into engineered SARS-CoV-2 vaccine vectors. As an initial proof-of-concept for heterologous glycoprotein display in the ΔE SARS-CoV-2 backbone, we evaluated genetic stability, structural integrity, functionality of the introduced glycoprotein, and the safety and immunogenicity of the resulting constructs.

The *VSV-G* gene was introduced into the safe, live ΔE SARS-CoV-2 backbone using the CLEVER reverse genetics system. Expression of VSV-G in infected host cells was confirmed, and the genetic stability of foreign sequences was validated for more than 10 passages. However, we observed that the genomic location of the integration site had a critical impact on viral fitness. Although integration sites were selected based on proven, stable insertions 5′ from the *N* gene and separated by a P2A self-cleavage peptide,^18^^,^^24^ or according to an earlier study on SARS-CoV,^20^ only ΔE^S+VSVG^ and ΔE^ΔS/VSVG^ produced high viral titers. These findings indicate that additional intrinsic properties of the nucleotide sequence may influence incorporation in the SARS-CoV-2 genomic background.

During serial passaging, recurrent mutations emerged in the cytoplasmic tail of spike in ΔE^S+VSVG^. The FEAR^∗^ mutation, or an alternative prolonged cytoplasmic tail, was detected in 8/10 rescued viral populations and was associated with higher viral titers, suggesting a potential replicative advantage. Related spike truncations have previously been shown to enhance its trafficking and incorporation into pseudotyped particles.^31^^,^^41^^,^^42^^,^^43^ We hypothesize that the FEAR^∗^ mutation in our example exerts a feature for the spatial convergence of spike and VSV-G at budding sites. Mechanistically, these deletions likely affect the ER-retention signal in the cytoplasmic tail, which would then lead to a redistribution of spike to other cellular compartments. A similar redistribution of glycoproteins from the ER/Golgi to the plasma membrane in a VSV-pseudotyped system has also been described for hantavirus.^44^ This phenomenon may depict an essential determinant for future efforts to co-express heterologous glycoproteins along with spike. As all detected variants carried distinct mutations, stable integration of a FEAR^∗^ mutation into the vaccine backbone would help stabilize the genetic background. Other recurrent mutations, such as F3677C in *ORF1ab* and G147R in *M* (in ΔE^ΔS/VSVG^), should additionally be integrated to achieve genetically stable populations.

Replacement of VSV-G with alternative glycoproteins may require additional evaluation, particularly when the heterologous glycoprotein exhibits lower fusogenicity than VSV-G. Under such conditions, co-expression with spike may be difficult to maintain due to selective pressure. Adaptation strategies, such as propagation on ACE2-negative cells, may help maintain stability under these conditions.

Structural analyses with cryo-EM confirmed successful incorporation of the heterologous glycoprotein, showing distinct VSV-G structures on the SARS-CoV-2 virion surface. ΔE^ΔS/VSVG^ particles closely resembled wild-type VSV, whereas ΔE^S+VSVG^ displayed heterogeneous surfaces with mixed clusters of VSV-G and spike, the former being more abundant. Clustering of two glycoproteins in VSV-based vectors^45^ may reflect lateral interactions of homologous membrane proteins.^46^ Immunogold labeling further supported these observations, revealing strong binding of VSV-G-directed antibodies. Functionality of the incorporated glycoprotein was demonstrated by efficient neutralization with α-VSV-G antibodies and by the broadened cellular tropism of both viral constructs. Incorporation of VSV-G enabled ACE2-independent cell entry, consistent with the receptor usage of VSV. This could confer a selective advantage over wild-type SARS-CoV-2, emphasizing the importance of strict biosafety measures such as the propagation-restricted ΔE constructs used here. Future vaccine candidates should incorporate enhanced safety features, e.g., by including mutated transcriptional regulatory sequences (TRS) to prevent any *in vivo* recombination or reversion to full replication.

Interestingly, the virus co-expressing VSV-G and spike was almost completely neutralized by α-VSV-G antibodies, making the independent contribution of spike difficult to assess experimentally. This could reflect reduced spike incorporation, steric hindrance caused by VSV-G-bound antibodies, or a lower functional contribution of spike to viral entry. In addition, the robust VSV-G-directed antibody responses observed *in vivo*, together with comparatively weaker spike responses, suggest the possibility of immunodominance by the heterologous glycoprotein in dual-expressing vectors. Such an effect could result from higher surface abundance of VSV-G, intrinsic differences in immunogenicity, or antigenic competition between co-displayed glycoproteins. While these observations do not affect the central proof-of-concept demonstrated here, they identify an important optimization challenge for future multivalent vector designs. Nevertheless, spike-specific antibody responses were detected, and spike-co-expressing constructs elicited stronger overall humoral responses following intranasal administration, supporting continued functionality of spike in the chimeric particles.

Both vaccine constructs were evaluated in two established animal models for SARS-CoV-2: Syrian hamsters and K18-hACE2 mice. Syrian hamsters are naturally susceptible to SARS-CoV-2, allowing efficient SARS-CoV-2 transmission, and are thus commonly used for safety and immunity studies, whereas K18-hACE2 mice ectopically express human ACE2 and develop severe, often lethal SARS-CoV-2 disease, making them a stringent model for evaluating live vaccine safety.^47^

Neither species exhibited body weight loss, viruses were rapidly cleared from the upper respiratory tract, and no transmission of vaccine virus was observed in hamsters, supporting the favorable safety profile of ΔE-based vectors. These findings are consistent with previous studies of the parental ΔE vaccine virus, suggesting that incorporation of VSV-G does not substantially alter the overall safety characteristics of the platform. Nevertheless, the expanded tropism conferred by VSV-G may require further investigation regarding tissue distribution and persistence at the injection site.

Antigen-specific antibody responses against VSV-G and, where present, spike were detected following a single immunization. Responses were observed in intranasally vaccinated hamsters and in intramuscularly vaccinated mice, whereas no substantial humoral response was measurable after intranasal immunization of K18-hACE2 mice. Similar observations have been reported previously, where intranasal vaccination of K18-hACE2 mice^48^^,^^49^ or cotton rats^50^ failed to induce strong antibody responses, whereas robust responses were observed after intramuscular vaccination or in intranasally vaccinated hamsters. Notably, a strong immune response could be achieved in K18-hACE2 mice following a booster immunization.^48^^,^^49^ Previous studies with the parental ΔE vector likewise demonstrated induction of neutralizing antibodies and complete protection following a prime-boost regimen, suggesting that booster vaccination may be required for optimal immunogenicity of the chimeric constructs. The present study was designed to evaluate vector stability, safety, and antigen expression after a single immunization; therefore, protective efficacy, durability of the immune response, and cellular immune responses remain to be addressed in future studies.

Future studies will also need to determine whether protection can be achieved in the absence of spike expression. This question is particularly relevant for the ΔE^ΔS/VSVG^ construct, where immunity against SARS-CoV-2 would rely entirely on non-spike antigens. Although spike-independent protective immunity has been documented,^51^ and the presence of numerous B and T cell epitopes across the SARS-CoV-2 proteome further supports this concept,^14^ it remains unclear whether a spike-deficient SARS-CoV-2 vector can induce protection sufficient for vaccine applications. Demonstration of such protection would substantially broaden the applicability of the platform by enabling complete replacement of spike with clinically relevant heterologous antigens while retaining SARS-CoV-2-directed immunity.

Previous studies have highlighted the importance of the interplay between viral glycoprotein expression, viral tropism, route of administration, and host receptor distribution as important determinants of vaccine performance.^30^^,^^34^^,^^48^^,^^49^^,^^50^ Accordingly, future studies should carefully consider these variables and include comparative analyses across relevant animal models. Pre-existing immunity will also need to be addressed, as for any live virus vaccine strategy; however, seroprevalence of α-VSV-G antibodies in humans is very low.^52^

Live vaccine viruses offer several advantages, including presentation of glycoproteins in their native membrane-anchored conformation, mimicking natural infection and facilitating efficient cell entry. The platform’s flexible design supports multiple routes of administration, enabling intranasal delivery to stimulate mucosal immunity as well as intramuscular administration for systemic immune responses. The ΔE design allows a single round of replication, enabling endogenous antigen amplification similar to mRNA vaccines while remaining propagation-restricted. In addition, the vector can be propagated to high titers under controlled conditions, facilitating cost-effective manufacturing. Importantly, the parental ΔE vaccine virus has already been downgraded to BSL-2 in three European countries, and we anticipate that similar regulatory assessments may allow production of future derivatives under BSL-2 conditions.

Taken together, our findings demonstrate that SARS-CoV-2 can stably incorporate and display a functional heterologous viral glycoprotein while maintaining genetic stability and a favorable safety profile. These results establish the ΔE SARS-CoV-2 backbone as a versatile platform for heterologous antigen delivery and viral vector engineering, and provide a foundation for future studies addressing protective efficacy, cellular immunity, and the incorporation of heterologous antigens.

### Limitations of the study

This proof-of-concept study shows the structural and functional incorporation of a heterologous glycoprotein into SARS-CoV-2 particles; however, *in vivo* studies were limited to preliminary safety and humoral antibody responses after a single immunization in Syrian hamsters and K18-hACE2 mice. Based on the small sample size per sex (2–3 animals per sex per group), sex as a biological variable was not assessed. However, the data revealed no obvious sex-related differences in immunogenicity outcomes.

Protective efficacy, durability of immune response, and cellular immune response could not be addressed within this study. Further, spike functionality on dual-expressing particles was not fully characterized, and the potential immunodominance of VSV-G remains to be addressed in future studies.

## Resource availability

### Lead contact

Further information and requests for resources and reagents should be directed to and will be fulfilled by the lead contact, David Hauser (david.hauser@unibas.ch).

### Materials availability

All plasmids generated in this study are deposited at Addgene and the European Virus Archive (EVA-Global). Viruses and cell lines for virus production are available through EVA-Global; we may require a completed materials transfer agreement if there is potential for commercial application. Individual accession numbers are provided with the key resources table.

### Data and code availability

All NGS data are available via NCBI: PRJNA1416039. All remaining data reported in this paper will be shared by the lead contact upon request. This paper does not report original code. Any additional information required to reanalyze the data reported in this paper is available from the lead contact upon request.

## Acknowledgments

Special thanks go to Thomas Klimkait for the continuous support throughout the project and the profound manuscript review. We thank Daniel Pinschewer, University of Basel, CH, and his team for providing the S309 and VI10 antibodies, the VSV Indiana isolate, and for granting access to their facilities for VSV-related experiments. We further thank Daniel for scientific input and comments on the manuscript. We thank mib Dienstleistung GmbH, GER, especially Martin Obermeier, Malik Prentice, and Özlem Yildirim, for support with NGS services. We are grateful to Carola Alampi from the BioEM Lab, University of Basel, CH, for assistance with electron microscopy. We also acknowledge the DBM Microscopy Core Facility, University of Basel, CH, particularly Ewelina Bartoszek-Kandler and Loïc Sauter, for their support with image acquisition and analysis. We are grateful to Frank Klipp, Steffen Kiepert, Christian Lipinski, and Felix Zimak for constant help in performing animal experiments and to Mareen Grawe for sample analysis. We thank G. Kochs, Freiburg, GER, for providing the SARS-CoV-2 Wuhan isolate. We thank Volker Thiel, University of Bern, CH, for providing Vero E6. We thank Sven Reiche, FLI, GER, for the generous provision of monoclonal α-S and α-N antibodies, which were obtained through the EU H2020 project EVA-GLOBAL, project #871029, and Yoichi Miyamoto, Nat. Inst. Biomed. Innovation, Health and Nutrition, Osaka, JP, for the α-ORF6 antibody. Graphical symbols in Figures 3, 4, S7, and in the graphical abstract were created with BioRender.com. The project was supported through a federal project grant through the Innosuisse project 119.403 IP-LS.

## Author contributions

Conceptualization, E.T.K., J.S., D. Hoffmann, F.O., and D. Hauser.; methodology, E.T.K., J.S., M.C., S.F., D. Hoffmann, and F.O.; validation, E.T.K. and J.S.; investigation, E.T.K., J.S., M.C., S.F., D. Hoffmann, F.O., and D. Hauser; resources, S.F.; data curation, E.T.K., F.O., and D. Hauser; writing – original draft, E.T.K.; writing – review and editing, J.S., S.F., M.B., D. Hoffmann, F.O., and D. Hauser; visualization, E.T.K. and D. Hauser; supervision, M.B., F.O., and D. Hauser; project administration, E.T.K., M.B., D. Hoffmann, F.O., and D. Hauser; funding acquisition, F.O. and D. Hauser.

## Declaration of interests

The authors declare no competing interests. The parental SARS-CoV-2 E-deletion backbone is covered by a patent family based on applications WO 203/036947 A1 and EP 26/153954.

## Declaration of generative AI and AI-assisted technologies in the writing process

During the preparation of this work, the authors used Grammarly and ChatGPT in order to check for readability and grammatical errors. After using this tool/service, the authors reviewed and edited the content as needed and take full responsibility for the content of the published article.

## STAR★Methods

### Key resources table

**Table:** 

REAGENT or RESOURCE	SOURCE	IDENTIFIER
**Antibodies**		

Murinized neutralizing α-SARS-CoV-Spike S309	Pinto et al.^53^	N/A
Murinized neutralizing α-VSV-G VI10	Kalinke et al.^54^	N/A
Mouse monoclonal α-β-actin	Cell Signaling Technology	Cat# 3700; RRID: AB_2242334; LOT# 20
Rabbit polyclonal α-SARS-CoV-2 NSP2	GeneTex	Cat# GTX135717; RRID: AB_2909866; LOT# B318853
Mouse monoclonal α-SARS-CoV-2 Nucleocapsid protein (4F3C4)	Bussmann et al.^55^	N/A
Sheep polyclonal α-SARS-CoV-2 ORF3a	Rihn et al.^56^	N/A
Rat monoclonal α-SARS-CoV-2 ORF6 (8B10)	Miyamoto et al.^57^	N/A
Mouse monoclonal α-SARS-CoV-2 ORF7a (3C9)	GeneTex	Cat# GTX632602; RRID: AB_2888320; LOT# 42219
Rabbit polyclonal α-SARS-CoV-2 ORF8	Novus Biologicals	Cat# NBP3-07972; LOT# 25966–2102
Mouse monoclonal α-SARS-CoV-2 Spike protein (4B5C1)	Gift from S. Reiche, FLI, Germany	N/A
Rabbit polyclonal α-VSV-G	Abcam	Cat# ab1874; LOT# 1032232–10
Rabbit polyclonal α-VSV-G (274/E)	This study	N/A
Donkey α-rat Cy3	Jackson ImmunoResearch	Cat# 712–165–153; RRID: AB_2340667
Donkey α-mouse Cy3	Jackson ImmunoResearch	Cat# 715–165–151; RRID: AB_2315777
Donkey α-rabbit Cy5	Jackson ImmunoResearch	Cat# 711–175–152; RRID: AB_2340607
Donkey α-sheep Cy5	Jackson ImmunoResearch	Cat# 713–175–147; RRID: AB_2340730
Donkey α-mouse HRP	Jackson ImmunoResearch	Cat# 715–035–151; RRID: AB_2340771
Donkey α-rabbit HRP	Jackson ImmunoResearch	Cat# 711–035–152; RRID: AB_10015282
Donkey α-sheep HRP	Jackson ImmunoResearch	Cat# 713–035–147; RRID: AB_2340710
Goat α-mouse Gold (6 nm)	Aurion	N/A

**Bacterial and virus strains**		

NEB 5α competent E. coli	Bioconcept	Cat# C2987H
Mycobacterium tuberculosis strain H37Rv	Gift from S. Gagneux, Swiss TPH	N/A
SARS-CoV-2 (Wuhan strain)	Gift from Georg Kochs, University of Freiburg, Germany	GenBank: OR018857
SARS-CoV-2 BetaCoV/Germany/BavPat1/2020	Gift from Bundeswehr Institute of Microbiology, Germany	GISAID accession: EPI_ISL_406862
VSV (Indiana Strain)	Gift from D. Pinschewer, University of Basel, Switzerland	N/A
VSV (Indiana Strain)	Gift from B. Tews, FLI, Germany	N/A
SARS-CoV-2 ΔE^S+VSV^^G^	This study	EVAg: 007V-06357 NCBI: PRJNA1416039; SRR37032437 and SRR37547943
SARS-CoV-2 ΔE^ΔS/VSV^^G^	This study	EVAg: 007V-06358 NCBI: PRJNA1416039; SRR37032436 and SRR37547942
SARS-CoV-2 ΔE^ΔE3a/VSV^^G^	This study	N/A
SARS-CoV-2 ΔE^VSV^^G-P2A^^^-^^^N^	This study	N/A
SARS-CoV-2 ΔE^esxA^	This study	EVAg: 007V-06361 NCBI: PRJNA1416039; SRR37547940 and SRR37547941

**Chemicals, peptides, and recombinant proteins**		

JAK-I inhibitor Pyridone 6 (CAS 457081–03–7)	Merck	Cat# 420097
NF-κB inhibitor QNZ (CAS 545380–34–5)	Merck	Cat# 481406
Albumin (Recombumin)	Sartorius	Cat# 205–005
Glutamine	Bioconcept	Cat# 5-10K50-H
Halt™ Protease and Phosphatase Inhibitor Cocktail	ThermoFisher	Cat# 78440
Hoechst 33342	Sigma-Aldrich	Cat# B2261
Phalloidin-iFluor488	Abcam	Cat# ab176753

**Critical commercial assays**		

NEBuilder® HiFi DNA Assembly	NEB	Cat# E2621
SF Cell Line 4D-Nucleofector-X Kit	Lonza	Cat# V4XC-2012
Maxwell® RSC Viral Total Nucleic Acid Purification Kit	Promega	Cat# AS1330
SuperScript IV One-Step RT-PCR System	Invitrogen	Cat# 12594100
qScript XLT One-Step RT-qPCR ToughMix	QuantaBio	Cat# 95132

**Deposited data**		

Next generation sequencing data	This paper	NCBI: PRJNA1416039

**Experimental models: Cell lines**		

Monkey: Vero E6 cells	Gift from V. Thiel, University of Bern, Switzerland	N/A
Monkey: Vero E6 cells (Vero C1008)	ATCC	Cat# CRL-1586
Monkey: Vero E6-TMPRSS2 cells	Lett et al.^36^	N/A
Monkey: Vero E2T cells	Lett et al.^36^	EVAg: 007S-06365
Monkey: Vero 76 cells	Collection of Cell Lines in Veterinary Medicine	CCLV Cat# CCLV-RIE 0228, RRID: CVCL_0603
Human: A549 AT cells	NIBSC	Cat# 101006
Human: HEK293T cells	Gift from D. Pinschewer, University of Basel, Switzerland	N/A
Human: HEK293T-indE cells	Lett et al.^36^	EVAg: 007S-06364
Human: CaCo-2 cells	Gift from G. Kochs, University of Freiburg, Germany	N/A
Human: HEp2	Sigma-Aldrich	Cat# 6803501
Hamster: BHK cells	Invitrogen	Cat# R700-01
Hamster: BSR T7/5 cells	Buchholz et al.^58^	CCLV Cat# CCLV-RIE 0583, RRID: CVCL_RW96
Hamster: CHO-K1 cells	Sigma-Aldrich	Cat# 85051005
Canine: MDCK cells	Sigma-Aldrich	Cat# 00062107

**Experimental models: Organisms/strains**		

Mouse: B6.Cg-Tg[K18-ACE2]2Prlmn/J	Charles River	RRID: IMSR_JAX:034860
Hamster: RjHan:Aura	Janvier Labs	N/A

**Oligonucleotides**		

For cloning primer sequences, please see Table S5	This study	N/A
nCoV_IP4-14059_Fw: GGTAACTGGTATGATTTCG	WHO/National Reference Center for Respiratory Viruses, Institut Pasteur, Paris, France^59^	N/A
nCoV_IP4-14146_Rv: CTGGTCAAGGTTAATATAGG		N/A
nCoV_IP4-14084_Probe(+): TCATACAAACCACGCCAGG [5′]Fam [3′]BHQ-1		N/A

**Recombinant DNA**		

Plasmid: pCMV-VSV-G	NovoPro	Cat# V010488
Plasmid: pCAGGS-VSG-IRES-Puro	Hitoshi et al.^60^	N/A
Plasmid: pD1	This study	RRID: Addgene_253115 EVAg: 007*N*-06352
Plasmid: pD1_XBB.1.5 S	Schön et al.^35^	RRID: Addgene_253119 EVAg: 007*N*-06318
Plasmid: pD2_ΔE673^∗^	Schön et al.^35^	RRID: Addgene_253186
Plasmid: pD2_ΔE 3^∗^Δ67a/VSVG	This study	RRID: Addgene_253171 EVAg: 007*N*-06353
Plasmid: pD_ΔS/VSVG ΔE67a 3^∗^	This study	RRID: Addgene_253173 EVAg: 007*N*-06354
Plasmid: pD_Δ3aE/VSVG Δ67a 3^∗^	This study	RRID: Addgene_253172 EVAg: 007*N*-06356
Plasmid: pD2_ΔE67a 3^∗^-VSVG-P2A-N	This study	RRID: Addgene_253170 EVAg: 007*N*-06355
Plasmid: pD2_ΔE/esxA Δ67a 3^∗^	This study	RRID: Addgene_253180
Plasmid: pCMV-esxA	This study	RRID: Addgene_253181

**Software and algorithms**		

Snapgene v8.0.3	Snapgene	https://www.snapgene.com/; RRID: SCR_015052
AresUI	Promega Maxwell	N/A
StepOne Software v2.3	Applied Biosystems StepOne	RRID: SCR_014281
ImmunoSpot® Analyzer Pro DC Software v7.0.38.15	CTL	RRID: SCR_011082
Porechop v0.2.4	Ryan Wick via github	https://github.com/rrwick/Porechop; RRID: SCR_016967
minimap2 v.2.28	Li^61^	https://github.com/lh3/minimap2; RRID: SCR_018550
BCFtools v1.21	Danecek et al.^62^	https://samtools.sourceforge.net/mpileup.shtml; RRID: SCR_005227
cuteSV v.21	Jiang et al.^63^	https://github.com/tjiangHIT/cuteSV; RRID: SCR_025233
BEDTools v2.31.1	Quinlan and Hall^64^	https://github.com/arq5x/bedtools2; RRID: SCR_006646
Fusion FX7 edge v18.09	Vilber	https://www.vilber.de/en/products/chemiluminescence/fusion-fx/
Nikon NIS Elements AR	Nikon	RRID: SCR_027181
GraphPad Prism v10	GraphPad Prism	RRID: SCR_002798
ImageJ v2.9.0/1.53t	NHI	https://imagej.nih.gov/ij/download.html; RRID: SCR_003070
Adobe Illustrator	Adobe	https://www.adobe.com/products/illustrator.html; RRID: SCR_010279
Omero	Open Microscopy Environment	https://www.openmicroscopy.org/omero/; RRID: SCR_002629

### Experimental model and study participant details

#### Animals

All procedures involving animals complied with institutional and national guidelines for the care and use of animals in research. The study protocol was approved by the relevant ethical review board (LVL MV TSD/7221.3-1-040/22).

##### Mice

Per group, three male and two female K18-hACE2 transgenic mice (*Mus musculus*, B6.Cg-Tg(K18-ACE2)2Prlmn/J; RRID: IMSR_JAX:034860), aged 6 to 9 weeks, were procured from Charles River Laboratories (Sulzfeld, Germany). The mice were housed in groups of up to three within individually ventilated cages (IVCs) and were provided with food and water *ad libitum*. Housing conditions were regulated at a temperature of 20°C–24°C and humidity levels of 45–65%, with a 12-h light/dark cycle that included a 30-min dawn transition.

##### Hamster

Specific pathogen-free (SPF) Syrian hamsters (*Mesocricetus auratus*; RjHan:AURA) were obtained from Janvier Labs (Le Genest-Saint-Isle, France). Three male and two female hamsters, aged 8 weeks, were used per group. They were housed in groups of up to three per IVC, maintained at a temperature of 20°C–24°C with 45–65% humidity. A 12-h light/dark cycle with a 30-min dawn and dusk transition was implemented. Standard rodent chow and water were provided *ad libitum*, along with enrichment materials such as straw and paper for nest building.

Following an acclimatization period, baseline measurements, including body weight and general health assessments, were recorded.

#### Cell lines

African green monkey kidney cells (Vero) were kindly provided by V. Thiel, Bern, Switzerland (Vero E6) or obtained from ATCC (Vero E6 [VERO C1008], Cat# CRL1586) and the Collection of Cell Lines in Veterinary Medicine (CCLV) (Vero 76 [CCLV_RIE 0228; RRID: CVCL_0603]). Adenocarcinomic human alveolar basal epithelial cells expressing ACE2 and TMPRSS2 (A549 AT) were obtained from NIBSC (A549-ACE-2 Clone 8-TMPRSS2; product number 101006). HEK293T cells were kindly provided by D. D. Pinschewer, University of Basel, Switzerland. CaCo-2 cells were kindly provided by G. Kochs, University of Freiburg, Germany. BHK cells were from Invitrogen, Thermofisher (Cat# R700-01), the derivative BSR T7/5 from CCLV (CCLV_RIE 0583; RRID: CVCL_RW96).^58^ CHO-K1 (Cat# 85051005), HEp2 (Cat# 6803501), and MDCK (Cat# 00062107) were purchased from Sigma-Aldrich. HEK293T-indE (HEK293T-E Tet:E-IRES-ORF6), Vero E2T, and Vero E6-TMPRSS2 (VeroTMP) cells were generated in-house as described before.^36^

Cells were cultivated in Dulbecco’s modified Eagle medium (DMEM), high-glucose media (Cat# 1-26F50-I, BioConcept) supplemented with 10′000 U/mL of penicillin, 10 mg/mL of streptomycin (P/S) (Cat# 4-01F00-H, BioConcept) and 10% fetal bovine serum (FBS) (Cat# S0615, Sigma-Aldrich) at 37°C in a humidified atmosphere with 5% CO_2_. Upon infection, cells were maintained in corresponding media with 2% FBS and cultured at 34°C. Cultures were routinely monitored for Mycoplasma contamination using the qPCR-based service from Eurofins Genomics.

#### Virus

Virus stocks of the SARS-CoV-2 Wuhan strain were provided by G. Kochs, University of Freiburg, Germany (SARS-CoV_FR-3 [GenBank OR018857]) and by the Bundeswehr Institute of Microbiology, Munich, Germany (SARS-CoV-2 Germany/BavPat1/2020 [GISAID accession EPI_ISL_406862]).

The Indiana strain of Vesicular Stomatitis Virus has been kindly provided by D. D. Pinschewer, University of Basel, Switzerland, and by B. Tews, Institute of Infectology, Friedrich-Loeffler-Institut, Greifswald, Germany.

All work involving infectious VSV was performed in registered facilities at the Department of Biomedicine, University of Basel, or at the Friedrich-Loeffler-Institut. Experiments with infectious SARS-CoV-2 and recombinant derivatives were conducted in biosafety level 3 (BSL-3) facilities at the same institutions.

### Method details

#### DNA fragments for the generation of recombinant SARS-CoV-2

Recombinant SARS-CoV-2 and its derivatives were generated as described before.^37^ In short, the genome was divided into four fragments (A-D) based on the reference sequence MT066156 (fragment A: nt1-8594; fragment B: nt8590-15107; fragment C: nt15100-20958; fragment D: nt20950-29867). Plasmids containing fragments A, B, and C were synthesized by GenScript. Fragment D was further divided into two fragments (D1: nt20950-25522 and D2: nt25422-29867) and cloned into a modified pUC19 plasmid backbone. Primers and PCR settings to produce fragments A, B, C, D, D1, and D2 are listed in.^37^

To reconstitute recombinant chimeric viruses, the *VSV-G* gene was cloned from plasmid pCMV-VSV-G (NovoPro; Cat# V010488) into the different genomic locations using NEBuilder® HiFi DNA Assembly (NEB; Cat# E2621). For proteomics experiments, *esxA* was cloned into pcDNA3.1 under the expression of a CMV promoter and was used as a positive control. Primers are listed in Table S5. All constructs are based on the “ΔE” backbone (deletion of the *E* gene, *ORF6*, *ORF7a*, and a premature stop codon at position 213 in *ORF3a* [ΔE67a 3^∗^]), and either fragment D or D2 was cloned. All plasmids are listed in Table S6.

To create pD2_ΔE67a 3^∗^-VSVG-P2A-N, VSVG-P2A was introduced using overlapping primer pairs F N-P2A/R VSVG-P2A and F VSVG/R 8-VSVG. To create pD2_ΔE 3^∗^Δ67a/VSVG, VSV-G was introduced with primer pairs F VSVG/R 7a-VSVG and F 7a-VSVG/R VSVG. The first 39 nucleotides (nt) and last 36 nt of *ORF7a* have been preserved in order to keep TRS integration regions. To create pD_Δ3aE/VSVG Δ67a 3^∗^, VSV-G was introduced with primer pairs F M/R VSVG-M and F VSVG-S/R S. To create pD_ΔS/VSVG ΔE67a 3^∗^, VSV-G was introduced with primer pairs F VSVG/R 1 ab-VSVG and F 3a-VSVG/R VSVG. To create pD2,_ΔE/esxA Δ67a 3^∗^, *esxA* was amplified from genomic Tuberculosis DNA (MTB H37Rv strain) using overlapping primer pairs F esxA-rE and R esxA-M.

#### Transfection and recovery of recombinant SARS-CoV-2

Virus was recovered by transfection of HEK293T-indE cells using the SF Cell Line 4D-Nucleofector-X Kit (Lonza; Cat# V4XC-2012) and the 4D-Nucleofector X Unit (Lonza) with pulse code DS-150 as described in.^36^ All-in-E plasmid was co-transfected.^36^

The next day, medium was exchanged to DMEM 2% FBS, 1% P/S, JAK-I inhibitor Pyridone 6 (CAS 457081–03–7) at a final concentration of 2 *μ*M, as well as the NF-κB inhibitor QNZ (CAS 545380–34–5) at 20 nM. Co-culture with Vero E2T cells was started 2 days post-transfection in T25 flasks.

#### Viral RNA extraction and cDNA conversion

Supernatant was harvested, filtered with a Millipore 0.20 *μ*m filter unit (Merck; Cat#SLLGX13), and RNA was extracted using the Maxwell® RSC Viral Total Nucleic Acid Purification Kit (Promega; Cat# AS1330) following the manufacturer’s protocol. Viral RNA was used directly as a template for RT-PCR using SuperScript IV One-Step RT-PCR System (Invitrogen; Cat# 12594100).

#### Sanger sequencing of recombinant viruses

RNA was extracted as described above, and the region of interest was amplified using specific primer pairs and the SuperScript IV One-Step RT-PCR System (Invitrogen; Cat# 12594100). The amplified region was directly sent for Sanger or ONT sequencing service at Microsynth AG, Balgach, Switzerland.

#### Replication kinetics

Vero E2T cells were seeded at 40% confluency in 6-well plates and cultured at 37°C, 5% CO_2_ in DMEM supplemented with 10% FBS, 1% P/S overnight. The medium was replaced by DMEM 2% FBS, 1% P/S prior to inoculation with chimeric SARS-CoV-2 or wild-type at an MOI of 0.01 in a total volume of 2 mL. Infections were set up in triplicate. 3 hpi, cell layers were washed with PBS, and 2 mL of DMEM 2% FBS, 1% P/S was added. 12, 24, 30, 48, 72, 96, and 144 hpi, 300 *μ*L of supernatant were collected. Samples were filtered through 0.20 *μ*m and stored at −80°C. To compensate for sample removal, 300 *μ*L DMEM 2% medium was added back to the well. The time-course experiment was done in 3 biological replicates, starting with the same input material. Infectious titers were measured by focus-forming assay (FFA).

#### High titer stocks

High titer stocks for *in vivo* studies were produced as follows: Vero E2T cells were seeded in T75 flask in DMEM 10% FBS, 1% P/S, grown overnight at 37°C to reach confluency. The next day, medium was changed to DMEM 2% FBS, 1% P/S, and virus was added at MOI 0.01 in a total of 6 mL medium. Flasks were incubated at 34°C. At 3 dpi, half of the medium was exchanged with fresh medium supplemented with 2% FBS, 1% P/S, 2 × glutamine (Bioconcept; Cat# 5-10K50-H), and 4 × 10^6^ Vero E2T cells per flask. Cultures were incubated for an additional 2 days. Supernatant was filtered through 0.20 *μ*m and stored at −80°C. Infectious viral particles were measured by FFA.

#### Replication on non-complementing cells

Replication of ΔE^S+VSVG^ and ΔE^ΔS/VSVG^ on non-complementing A549 AT cells was compared to wild-type SARS-CoV-2. Cells were seeded at 40% confluency in DMEM 10% FBS, 1% P/S, and grown overnight at 37°C. The next day, medium was removed, and virus was added at MOI 0.5 in 0.5 mL DMEM 2% FBS, 1% P/S. 3h post-infection, medium was removed, and the cell layer was washed with PBS. 1 mL DMEM 2% FBS, 1% P/S was added and incubated for 3 or 4 days. Supernatant was filtered through 0.20 *μ*m, and fresh cells were infected at a 1:100 dilution. The protocol was repeated every 3 or 4 days. Samples were analyzed by FFA.

#### Tropism

HEK293T, A549 AT, Vero E6, BHK-21, MDCK, CHO-K1, CaCo2, and HEp-2 cells were seeded at 80% confluency on coverslips in 24-well plates. Virus (ΔE^S+VSVG^, ΔE^ΔS/VSVG^, wild-type) was added at MOI 0.05 and incubated for 24 h in DMEM 2% FBS, 1% P/S. Infection events were analyzed by FFA.

#### Neutralization assay

Vero E6 cells were seeded in 96-well flat-bottom plates, 3.5 × 10^6^ cells/plate in a final volume of 100 *μ*L DMEM complemented with 2% FBS, 1% P/S. Cells were incubated at 37°C and 5% CO_2_ overnight to reach confluency. Murinized neutralizing α-SARS-CoV S cross-reactive antibody S309^53^ and murinized α-VSV-G antibody VI10^54^ were serially diluted 1:2 in a 96-well round-bottom plate, starting with 10 *μ*g/mL.

Virus was added to the diluted antibody and incubated for 1 h at 34°C, 5% CO_2_. Pre-incubated antibody/virus mix was added to the cells and incubated for 3 days at 34°C, 5% CO_2_. Cells were fixed in 4% PFA and stained with crystal violet. Images were acquired using a CTL Immunospot Analyzer, and neutralization capacity was assessed by comparing plaque size and number.

For the analysis of single infection events, VeroTMP cells were seeded in a 24-well plate format on coverslips and grown overnight at 37°C, 5% CO_2_ to reach confluency. In a separate plate, virus was added in DMEM 2% FBS, 1% P/S, and α-VSV-G (VI10) or α-VSV-G + α-S (S309) were added at 1:16, 1:64, 1:512, or 1:2048, starting from 10 *μ*g/mL each. Antibody/virus mix was incubated for 1 h at 37°C, 5% CO_2,_ before being added to the cells. Cells were incubated for 24 h at 34°C, 5% CO_2,_ before being fixed in 4% PFA and stained for N detection.

#### Next-generation sequencing

NGS was performed at mib Dienstleistung GmbH (Medizinisches Infektiologiezentrum Berlin) using a MinIon MK1D with a Flowcell (FLO-MIN114). The Library Prep Kit (Oxford Nanopore, SKU: EXP-MRT001) was used according to the manufacturer’s protocol. The Native Barcoding Kit 24 V14 (Oxford Nanopore) was used to barcode samples according to the manufacturer’s protocol.

Bioinformatic analysis was performed using an automated workflow. Raw sequencing data were processed with Porechop (v0.2.4) for adapter removal and demultiplexing. Reads were first aligned to the Wuhan-Hu-1 reference genome (MN908947.3) with minimap2 (v2.28)^61^ for filtering. These purified reads were then re-aligned to specific reference genomes for variant analysis using bcftools (v1.21)^62^ for SNPs/InDels and cuteSV (v1.21)^63^ for structural variants. Finally, a consensus sequence was generated, and low-coverage regions were masked using bedtools (v2.31.1).^64^

#### Immunoblotting

Vero E6 cells were seeded at confluency in 6-well plates and infected in DMEM 2% FBS, 1% P/S. 24 h post-infection, cell layers were washed with PBS and lysed in RIPA buffer supplemented with Halt Protease and Phosphatase Inhibitor Cocktail (ThermoFisher; Cat# 78440). Lysates were centrifuged at 16′000g and 4°C for 10 min, and supernatant was boiled at 95°C for 5 min, mixed with Laemmli buffer, and loaded onto a polyacrylamide gel (Bio-Rad; Cat# 4561094).

Proteins were analyzed by immunoblot using the Fusion FX imaging system. Antibodies are listed below.

#### Immunocytochemistry

For detection and quantification of infectious viral particles (focus forming assay, FFA) or protein expression analysis, approximately 1.2 × 10^5^ VeroTMP cells were seeded onto glass coverslips in 24-well plates and grown overnight at 37°C, 5% CO_2_ in DMEM 10% FBS, 1% P/S. Medium was changed to DMEM 2% FBS, 1% P/S, and cells were infected with virus variants in 500 *μ*L and incubated overnight. Cells were fixed with 4% PFA in PBS for 10 min at RT, washed, and subsequently stained. Briefly, cells were blocked with 10% Normal Donkey Serum (Cat# 017–000–121, Jackson ImmunoResearch) and 0.1% Triton X-100 for 1 h, followed by incubation with primary antibodies for 1 h in 1% Normal Donkey Serum, 1% BSA, and 0.3% Triton X-100 in PBS. Cells were washed three times for 10 min with 1× PBS, 0.1% BSA, and incubated with secondary antibodies for 1 h in 1% Normal Donkey Serum, 1% BSA, and 0.3% Triton X-100 in PBS. Cells were washed once with 1× PBS, 0.1% BSA, and washed three times with 1× PBS before mounting on microscope slides using Fluoromount-G (Cat# 0100-01, SouthernBiotech). Phalloidin-iFluor488 was co-applied with secondary antibodies to label F-actin (Abcam; ab176753). Hoechst 33342 dye (Sigma-Aldrich; Cat# B2261) was co-applied during washing at a final concentration of 0.5 *μ*g/mL for nuclear staining.

Microscopy images were acquired on a widefield microscope (Nikon Ti2 equipped with a Photometrics 95B camera, Nikon NIS AR software, using a 20× or 40× Plan-Apochromat objective [numerical aperture 0.75 or 0.95, respectively]), and were processed in Fiji and Omero. For infected foci quantification (focus-forming assay), images were analyzed with QuPath.^65^

#### Antibodies

The following antibodies were used in this study: mouse monoclonal anti-*β*-actin (Cell Signaling Technology; Cat# 3700; RRID: AB_2242334; LOT# 20), rabbit polyclonal anti-SARS-CoV-2 NSP2 (GeneTex; Cat# GTX135717; RRID: AB_2909866; LOT# B318853), mouse monoclonal anti-SARS-CoV-2 nucleocapsid protein (4F3C4, gift from Sven Reiche^55^), sheep polyclonal anti-SARS-CoV-2 ORF3a,^56^ rat monoclonal anti-SARS-CoV-2 ORF6 (8B10, gift from Y. Miyamoto,^57^ mouse monoclonal anti-SARS-CoV-2 ORF7a (3C9; GeneTex; Cat# GTX632602; RRID: AB_2888320; LOT# 42219), rabbit polyclonal anti-SARS-CoV-2 ORF8 (Novus Biologicals; Cat# NBP3-07972; LOT# 25966-2102), mouse monoclonal anti-SARS-CoV-2 spike protein (4B5C1, gift from Sven Reiche), rabbit polyclonal anti-VSV-G (Abcam; Cat# ab1874; LOT# 1032232-10).

Fluorophore-conjugated or peroxidase-conjugated secondary antibodies were from Jackson ImmunoResearch (Cy3 donkey anti-rat #712–165–153, RRID: AB_2340667; Cy3 donkey anti-mouse #715–165–151, RRID: AB_2315777; Cy5 donkey anti-rabbit #711–175–152, RRID: AB_2340607; Cy5 donkey anti-sheep #713–175–147, RRID: AB_2340730; HRP donkey anti-mouse #715–035–151, RRID: AB_2340771; HRP donkey anti-rabbit #711–035–152, RRID: AB_10015282; HRP donkey anti-sheep #713–035–147, RRID: AB_2340710).

#### Mice inoculation

Mice were either intranasally (25 *μ*L/animal) or intramuscularly (50 *μ*L/animal) inoculated with ΔE^S+VSVG^ or ΔE^ΔS/VSVG^ under isoflurane-induced short-acting anesthesia. Virus stocks were dosed according to infectious units determined by focus-forming assay before inoculation (5.25 × 10^4^ infectious units/animal). Back-titration after completion of the animal experiments yielded titers of approximately 10^3.07^–10^3.625^ TCID_50_ per inoculum. Animals were monitored daily for health status and clinical signs throughout 14 days, and on day 21 post-inoculation for weight changes.

To evaluate shedding, a combined swab of buccal cavity and nasal surface was taken with a moistened swab at 0, 1, 3, 7, 12 dpim and stored in 1 mL medium. Blood samples were either taken at the end timepoint (21 dpim) or by puncturing the *V. facialis* at day 0 for serological evaluation. At necropsy organ samples of nose, lung (caudal, medial, cranial), cerebrum, and cerebellum were taken for viral load assessment.

#### Hamster inoculation

The hamsters (*n* = 5 per group) were vaccinated intranasally using a volume of 70 *μ*L with ΔE^S+VSVG^ or ΔE^ΔS/VSVG^. Virus stocks were dosed according to infectious units determined by focus-forming assay before inoculation (5.25 × 10^4^ infectious units/animal). Back-titration after completion of the animal experiments yielded titers of approximately 10^3.07^–10^3.625^ TCID_50_ per inoculum. To evaluate potential transmission of the live vaccine, the ΔE^S+VSVG^-vaccinated animals were co-housed with serologically naive direct contact hamsters in a 1:1 setup, after separating them for 24 h following vaccination to exclude nonspecific transmission of the inoculum.

Throughout 14 days post-inoculation, and on day 21, animals were monitored daily for clinical signs, weight changes, and overall health status. Samples were collected at predetermined timepoints and included blood for serological analysis, nasal washing samples, and final organ samples of nose, lung (superior, medial, inferior), cerebrum, and cerebellum for viral load assessment.

Nasal washing samples were taken at 0, 1, 3, 7, 12 dpim by applying 200 *μ*L of PBS in each nostril and collecting the reflux under short isoflurane inhalation anesthesia. Blood samples were either taken at the end timepoint (21 dpim) or by puncturing the *V. saphena* at day 0 for serological evaluation.

#### Genome copy quantification by RT-qPCR (*in vivo* experiments)

A total of 100 *μ*L of nasal washing, nasal-buccal swab eluate, or organ sample supernatant (0.1 × 0.1 × 0.1 cm in 1 mL cell culture medium, a stainless-steel bead, ruptured in a tissue lyzer) was utilized for the extraction of nucleic acids, employing the NucleoMag Vet kit (Macherey-Nagel). To quantify SARS-CoV-2-specific nucleic acids, the RdRp locus was targeted.^59^ To confirm successful RNA extraction, the housekeeping gene *β-actin* was used. The RT-qPCR reactions were prepared using the qScript XLT One-Step RT-qPCR ToughMix (QuantaBio) in a total volume of 12.5 *μ*L, including 1 *μ*L of the FAM mix and 2.5 *μ*L of extracted RNA. The amplification was performed on a BioRad real-time CFX96 detection system (Bio-Rad) under the following cycling conditions: 50°C for 10 min, denaturation at 95°C for 1 min, followed by 42 cycles of 10 s at 95°C, 10 s at 60°C, and 20 s at 68°C. Fluorescence was measured during the annealing phase.

#### ELISA

Medium-binding plates (Greiner Bio-One GmbH) were coated with 100 ng/well of either the RBD antigen^66^ overnight at 4°C in 0.1 M carbonate buffer (pH 9.6) or were treated with coating buffer only. Thereafter, plates were blocked for 1 h at 37°C using 5% skim milk in PBS. Sera were incubated on the coated and uncoated wells for 1 h at room temperature. A multi-species conjugate (SBVMILK; obtained from ID Screen® Schmallenberg virus Milk Indirect ELISA; IDvet) was diluted 1:80 and then added for 1 h at room temperature. Following the addition of tetramethylbenzidine (TMB) substrate (IDEXX), the ELISA readings were taken at a wavelength of 450 nm on a Tecan Spectra Mini instrument (Tecan Group Ltd). Between each step, the plates were washed three times with TBST. The absorbance was calculated by subtracting the optical density (OD) measured on the uncoated wells from the values obtained from the protein-coated wells for the respective sample.

#### Virus neutralization assay (animal experiments)

Virus neutralization assay was conducted in a 96-well plate format. 100 *μ*L of pre-diluted sera (1:16) in triplicate per sample were added in row A, and 50 *μ*L of medium were prearranged in rows B-H. Serial 1:2 transfers were performed by moving 50 *μ*L from row to row (row A to H), then discarding 50 *μ*L from the final row, leaving 50 *μ*L per well. The respective virus stock (VSV Indiana or SARS-CoV-2 BetaCoV/Germany/BavPat1/2020) was adjusted to contain 100 TCID_50_/50 *μ*L. 50 *μ*L of the working virus suspension was added to all sample wells, including the positive and negative controls, and plates were incubated for 1 h at 37°C. In addition, five 10-fold dilutions were prepared to confirm the virus stock titer. Cells (Vero E6 cells for SARS-CoV-2 and Vero 76 cells for VSV) were detached, a confluent flask was resuspended in 10 mL medium, and 100 *μ*L cell suspension was dispensed per well. Plates were incubated for 3 days at 37°C. Finally, the cytopathic effect (CPE) was evaluated by light microscopy. The virus neutralization titer was then calculated by the formula: x=ab+−(log2c). The number of cell culture wells without virus replication was (a) divided by the number of cell culture wells per serum dilution (b) plus the -log_2_ of the pre-dilution of the sample (c).

#### Immunofluorescent antigen detection

For expression of VSV-G protein, BSR T7/5 cells^58^ were transfected with the pCAGGS^60^ based plasmid pCAGGS-VSVG-IRES-Puro, encoding the VSV Indiana strain *G* gene, followed by the encephalomyocarditis virus internal ribosome entry site (IRES) and a puromycin resistance gene. BSR T7/5 cells grown in T25 tissue culture flasks were split 1:1.3 into new T25 flasks. Three hours after seeding, the culture medium was replaced with 3 mL serum-free DMEM. For each T25 flask, 18 *μ*g plasmid DNA and 27 *μ*L polyethyleneimine (PEI; 1 *μ*g/*μ*L in ddH_2_O; Sigma-Aldrich) were each diluted in 1.2 mL DMEM, incubated separately for 5 min at room temperature, combined, and incubated for an additional 20 min. One hour after medium exchange, the transfection mixture was added to the cells. After 4 h, the transfection medium was removed, cells from two T25 flasks were trypsinized and resuspended in 21 mL complete culture medium, and 100 *μ*L of the cell suspension was transferred per well into 96-well tissue culture plates. At 24 h post-transfection, cells were fixed with 4% PFA and processed for immunofluorescence (IF) analysis. For detection and IF control of VSV-G expression, a polyclonal rabbit serum raised against purified VSV Indiana virions (α-VSV 274/E; 1:1000 in PBS) was used.

#### Cryo-transmission electron microscopy

Virus was grown in optiMEM medium (Gibco; Cat# 31985070) supplemented with 0.4% Albumin (Recombumin, Sartorius; Cat# 205-005) and 1% P/S. Viral supernatant was filtered using a Millipore 0.20 *μ*m filter unit (Merck; Cat# SLLGX13). All incubation steps were performed on a shaker at room temperature. Primary antibodies (murinized S309 or VI10, see above) were added at 1:20 and incubated for 2.5 h. A 6 nm gold-conjugated goat α-mouse secondary antibody (Aurion, The Netherlands) was added 1:5 and incubated for 6 h at room temperature. Virus particles were fixed in 1.8% glutaraldehyde (ThermoScientific; Cat# 233281000). A 4 *μ*L aliquot of sample was adsorbed onto a holey carbon-coated grid (Lacey, Tedpella, USA), blotted with Whatman 1 filter paper, and vitrified into liquid ethane at −180°C using a Leica GP2 plunger (Leica microsystems, Austria). Frozen grids were transferred onto a Talos 200C Electron microscope (FEI, USA) using a Gatan 626 cryo-holder (GATAN, USA). Electron micrographs were recorded at an accelerating voltage of 200 kV using a low-dose system (40 e-/Å2) and keeping the sample at −175°C. Micrographs were recorded on a 4 K × 4 K Ceta CMOS camera at a nominal magnification of 92′000 ×, corresponding to a pixel on the image of 1.6 Å. Defocus values were −2 to 3 *μ*m.

#### Proteomic analysis

VeroTMP cells were seeded at a density of 2 × 10^5^ cells per well 1 day before infection in a 12-well plate format. Cells were infected with an MOI of 0.1 with ΔE^esxA^. 24 h after infection, cells were washed twice in cold PBS and lysed in 2M guanidine hydrochloride, 0.1 M triethylammonium bicarbonate, and 10 mM TCEP. Samples were heated for 10 min at 95°C followed by 10 min sonication (30 s on, 30 s off per cycle) on a Pixul® system (Active Motif) using the following settings: Pulse [N]: 50; PRF [kHz]: 1; burst rate [Hz]: 20. Protein concentration was measured, extracts were alkylated using 20 mM iodoacetamide, and samples were digested with sequencing-grade modified trypsin (1/50, w/w; Promega, Madison, Wisconsin). Digested peptides were then purified with C18 reverse-phase spin columns (Macrospin, Harvard Apparatus) according to the manufacturer’s instructions and dried under vacuum.

Peptides were resuspended in 0.1% aqueous formic acid, and 0.2 *μ*g of peptides were subjected to PRM analysis using an Orbitrap Eclipse Mass Spectrometer fitted with a Vanquish *Neo* (both Thermo Fisher Scientific) and a custom-made column heater set to 60°C. Peptides were resolved using an RP-HPLC column (75 *μ*m × 30 cm) packed in-house with C18 resin (ReproSil-Saphir C18–AQ, 1.5 *μ*m resin; Dr. Maisch GmbH) at a flow rate of 0.2 *μ*Lmin^-1^. Separation of peptides was achieved using the following gradient: 2% Buffer B to 8% Buffer B in 5 min, 8% Buffer B to 25% Buffer B in 45 min, 25% Buffer B to 35% Buffer B in 10 min. Buffer A was 0.1% formic acid in water, and buffer B was 80% acetonitrile, 0.1% formic acid in water.

The mass spectrometer was operated in targeted acquisition mode. For the MS2 scans, the resolution of Orbitrap was set to 120K, the maximum injection time was 246 ms, and the Absolute AGC Target was set to 100000. The isolation window was set to 0.4m/z. The targeted peptide was LAAAWGGSGSEAYQGVQQK (636.646+++). A full MS1 was included (120k resolution, 300–1600 m/z scan range.

### Quantification and statistical analysis

Data were analyzed using GraphPad Prism 10 software. No inferential statistical tests were performed. Data are presented as descriptive statistics, and detailed definitions of n for each figure panel are provided in the figure legends. No statistical methods were used to pre-determine sample sizes.

## Supplemental information

Document S1. Figures S1–S8

Table S1. Region of interest sequencing results from recombinant viruses Cell-free supernatant from different passaging steps was analyzed.

Table S2. Analysis of ΔE(S + VSVG) for the detection of the FEAR^∗^ mutation Initial rescue populations (top) are shown, in which the FEAR^∗^ mutation was first observed. For replicate #1, no low-passage population was available. Additional replicates (bottom) were generated by transfecting four cultures, each split at 1 day post-transfection to obtain eight replicates. Populations were sequenced at passage 0 (p0) and after prolonged passaging (p > 3) following transfer of cell-free supernatant. Detected mutations are indicated and sequencing results of region of interest (ROI) are included. n.d., not determined (no stocks available); low titers, delayed CPE, no amplification possible.

Table S3. Summary of next-generation sequencing results for the recombinant viruses ΔE(S + VSVG) and ΔE(ΔS/VSVG) Two independent replicates (#1 and #2) are shown. For replicate #1, passage 1 (p1) and p3 are presented. For replicate #2, two populations from p0 (8 and 14 days post-transfection, dpt) and p1 are included. An “x” indicates a single nucleotide polymorphism (SNP) at the specific genomic position. Mutations detected across multiple populations are highlighted in blue; their frequencies (in %) and the corresponding amino acid changes are indicated. Positions also detected in high-passage stocks are highlighted in yellow. Position 20949 has been deliberately mutated to introduce a SalI restriction site.

Table S4. Sequencing results for the recombinant virus ΔE(esxA), related to Figure S8 Cell-free supernatant from different passaging steps was analyzed.

Table S5. List of primers used to clone VSV-G or TB-esxA into the ΔE background, related to STAR Methods

Table S6. Plasmids used for recombinant virus rescue, related to STAR Methods
