# Supplementary material for: Chimeric SARS-CoV-2 vaccine vectors stably incorporating the functional glycoprotein of VSV
Source: iScience. 2026 Sep 16;29(10):117521. doi: 10.1016/j.isci.2026.117521 (PMC13599654; doi:10.1016/j.isci.2026.117521)
Supplement: Supplementary file 1 [file mmc1.pdf]

iScience, Volume *29*

Supplemental information

Chimeric SARS-CoV-2 vaccine vectors stably incorporating the functional glycoprotein of VSV

Enja Tatjana Kipfer, Jacob Schön, Mohamed Chami, Stefan Finke, Martin Beer, Donata Hoffmann, Fabian Otte, and David Hauser

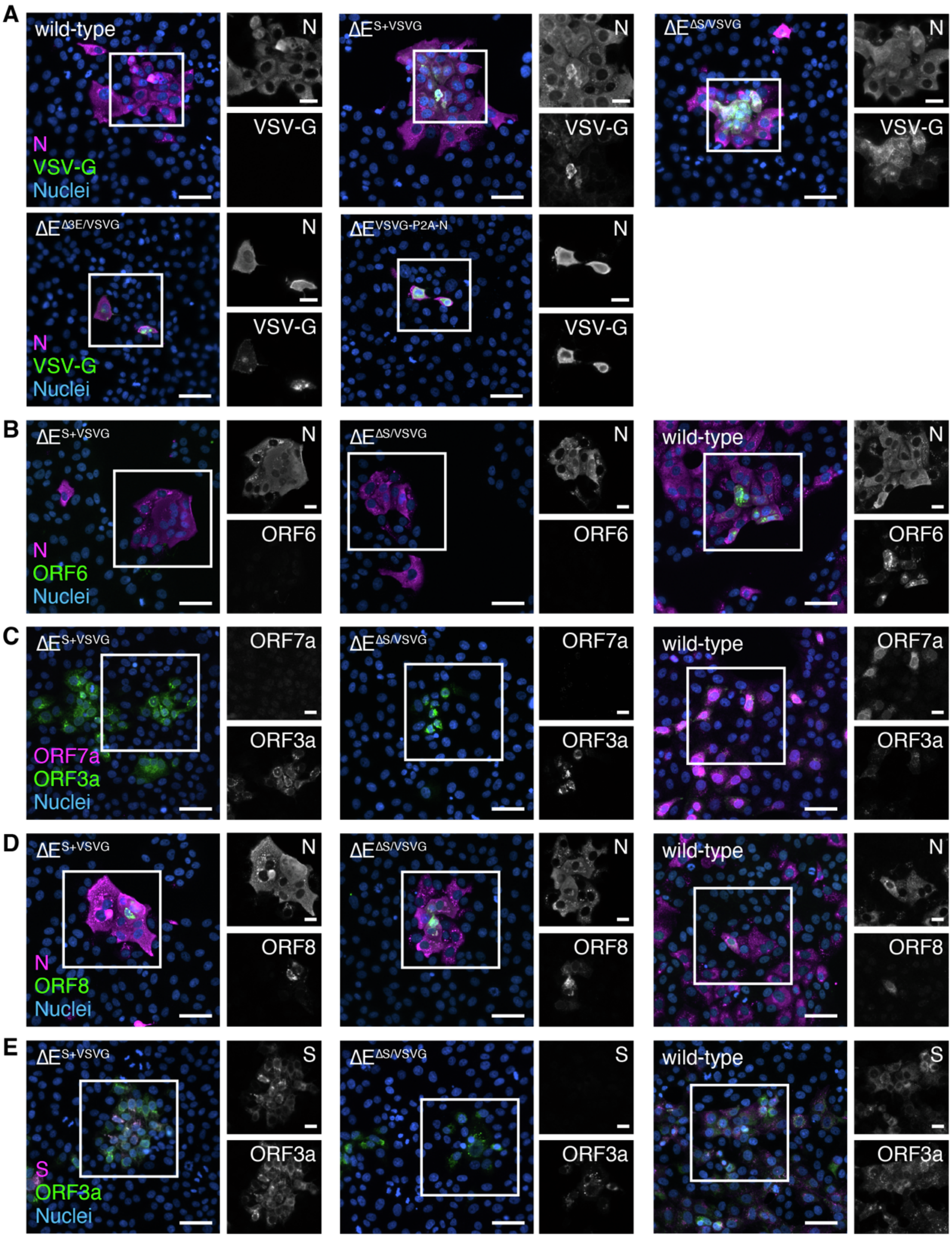

Figure S1. Viral protein detection by immunofluorescence, related to Figure 1

(**A**) Immunofluorescence staining of VeroTMP cells infected with low-passage ΔE^{S+VSVG}, $\Delta E^{\Delta S/VSVG}$, $\Delta E^{\Delta 3E/VSVG}$, $\Delta E^{VSVG\text{-}P2A\text{-}N,}$ and wild-type SARS-CoV-2 control for the detection of N (magenta) and VSV-G (green).

(**B-E**) Detection of viral proteins in VeroTMP cells infected with ΔE^{S+VSVG}, $\Delta E^{\Delta S/VSVG}$, and wild-type SARS-CoV-2 with (**B**) N (magenta) and ORF6 (green), (**C**) ORF7a (magenta) and ORF3a (green), (**D**) N (magenta) and ORF8 (green), or (**E**) S (magenta) and ORF3a (green) detected. Nuclei are in blue. Scale bar is 50 *μ*m in overview and 20 *μ*m in ROI images.

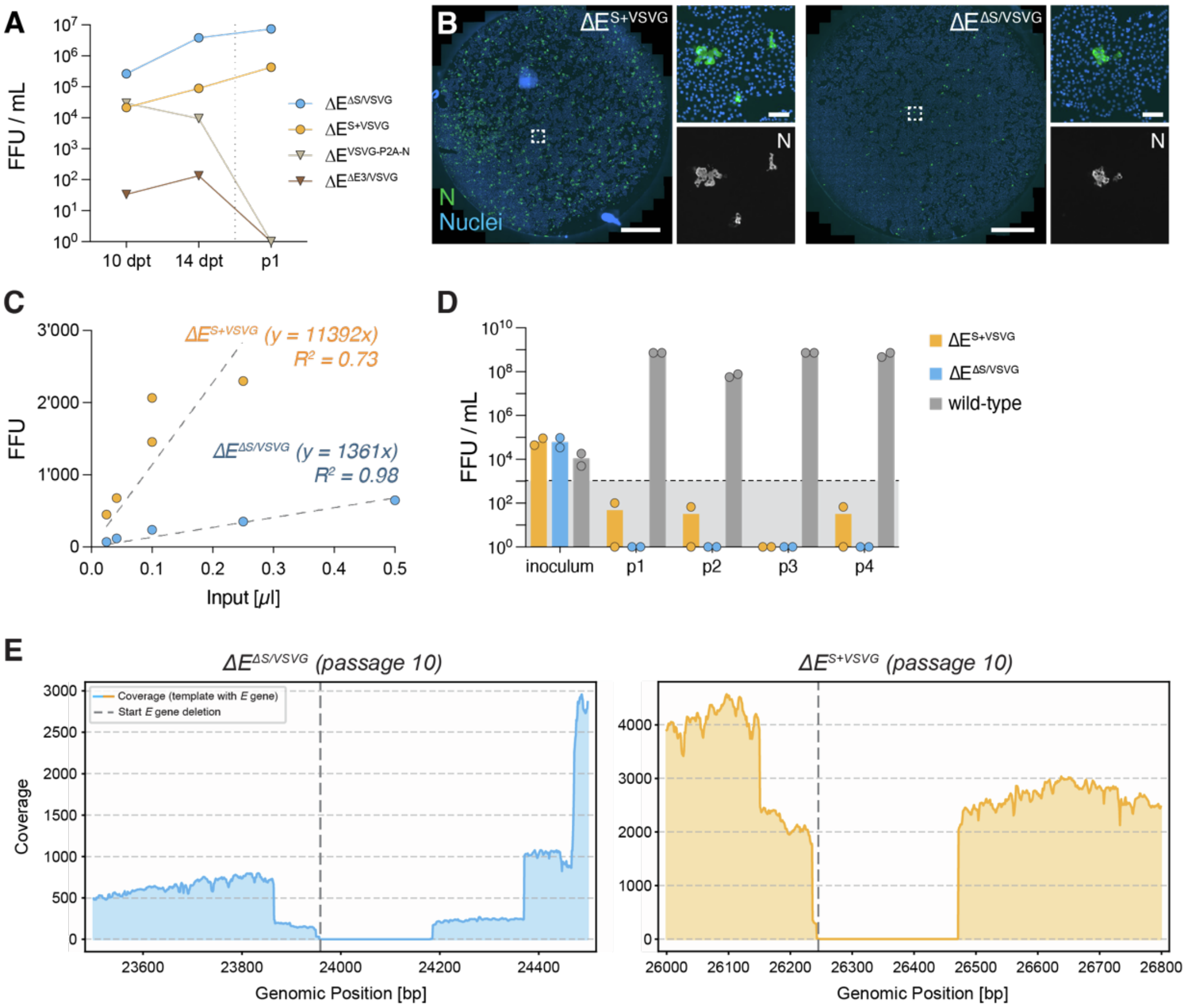

Figure S2. Supplemental characterization of chimeric viruses, related to Figures 1, 3, and 4

(**A**) Passaging of ΔE^{S+VSVG}, $\Delta E^{\Delta S/VSVG}$, $\Delta E^{VSVG\text{-}P2A\text{-}N}$, $\Delta E^{\Delta E3/VSVG}$. Titers were examined by FFA from representative images 10 and 14 days post-transfection (dpt) and after cell-free supernatant transfer to fresh VeroE2T cells (passage 1, p1).

(**B**) Representative example of infected cells to quantify viral titers for ΔE^{S+VSVG} and $\Delta E^{\Delta S/VSVG}$ in VeroTMP cells; detection of N is shown in green, nuclei are in blue. Scale bar is 2 mm in overview and 100 μm in ROI images.

(**C**) Titration of ΔE^{S+VSVG} and $\Delta E^{\Delta S/VSVG}$ and quantification by FFA. Linear fit and correlation are indicated for titer determination used to inoculate animals for *in vivo* studies. *n* = 2 technical replicates per dilution across 5 dilutions per virus.

(**D**) Replication competence of ΔE^{S+VSVG} and $\Delta E^{\Delta S/VSVG}$ in non-complementing A549 AT cells compared to wild-type SARS-CoV-2. Infectious particle production at each passage was quantified by FFA (*n* = 2 technical replicates*).* Detection limit at 10^3 FFU/mL; bar graphs represent the mean.

(**E**) Coverage plots showing read depth across the *E* gene deletion site. Reads were mapped to a reference genome containing the *E* gene. The dashed line indicates the start of the deletion.

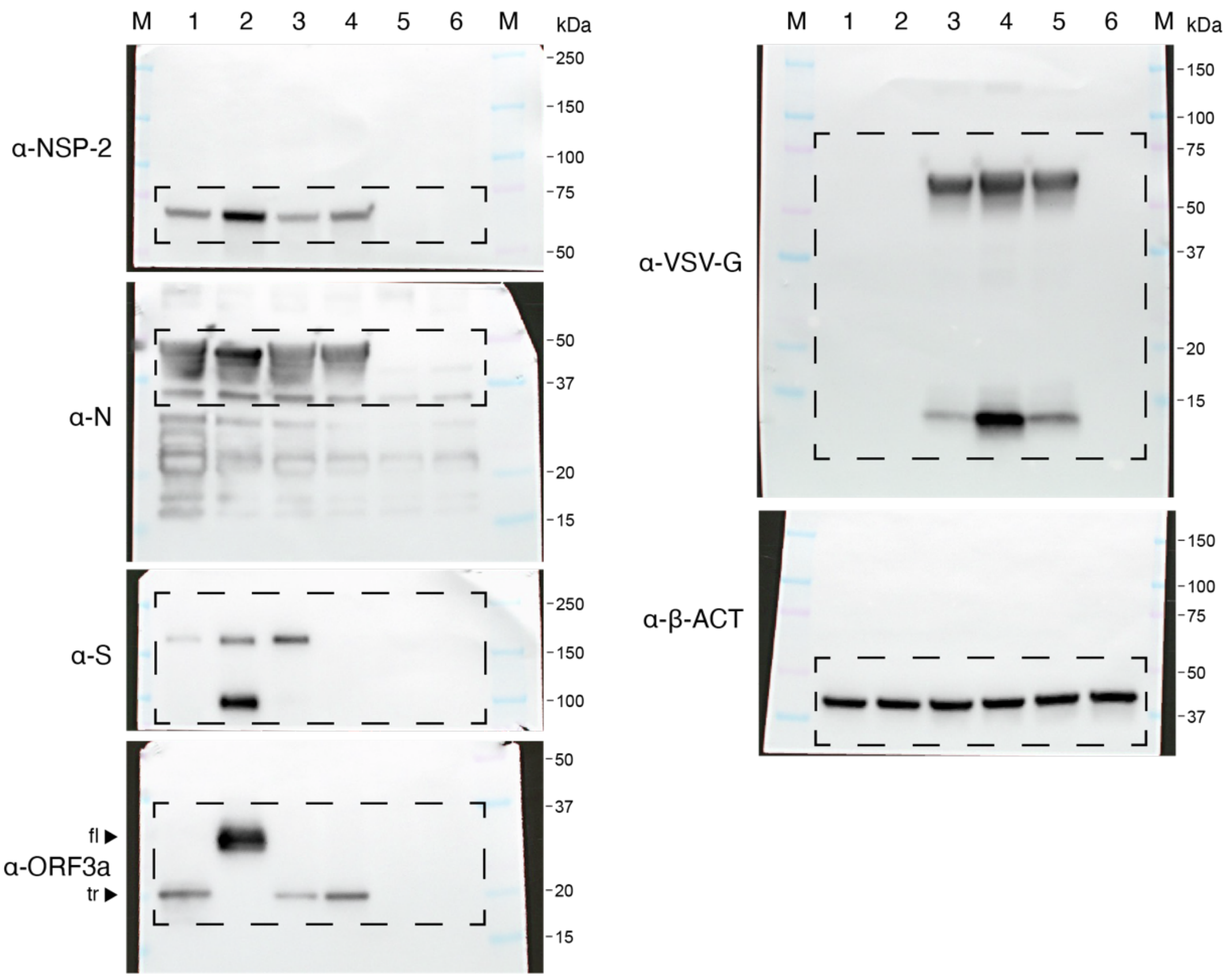

Figure S3. Uncropped immunoblot images, related to Figure 1

Uncropped immunoblot membranes corresponding to **Figure 1C**. Dashed boxes indicate the regions displayed in **Figure 1C**. Lanes are loaded as follows: 1, ΔE vaccine virus; 2, wild-type SARS-CoV-2; 3, ΔE^{S+VSVG}; 4, $\Delta E^{\Delta S/VSVG}$; 5, VSV-G plasmid transfection (positive control for VSV-G expression); 6, untransfected, uninfected VeroTMP cells (negative control). Antibodies used for detection are indicated on the left. Molecular weight markers (M, kDa) are shown on both sides of each membrane. *β*-actin (*β*-ACT) served as a cellular loading control. fl, full-length ORF3a; tr, truncated $ORF3a^{213*}$ variant.

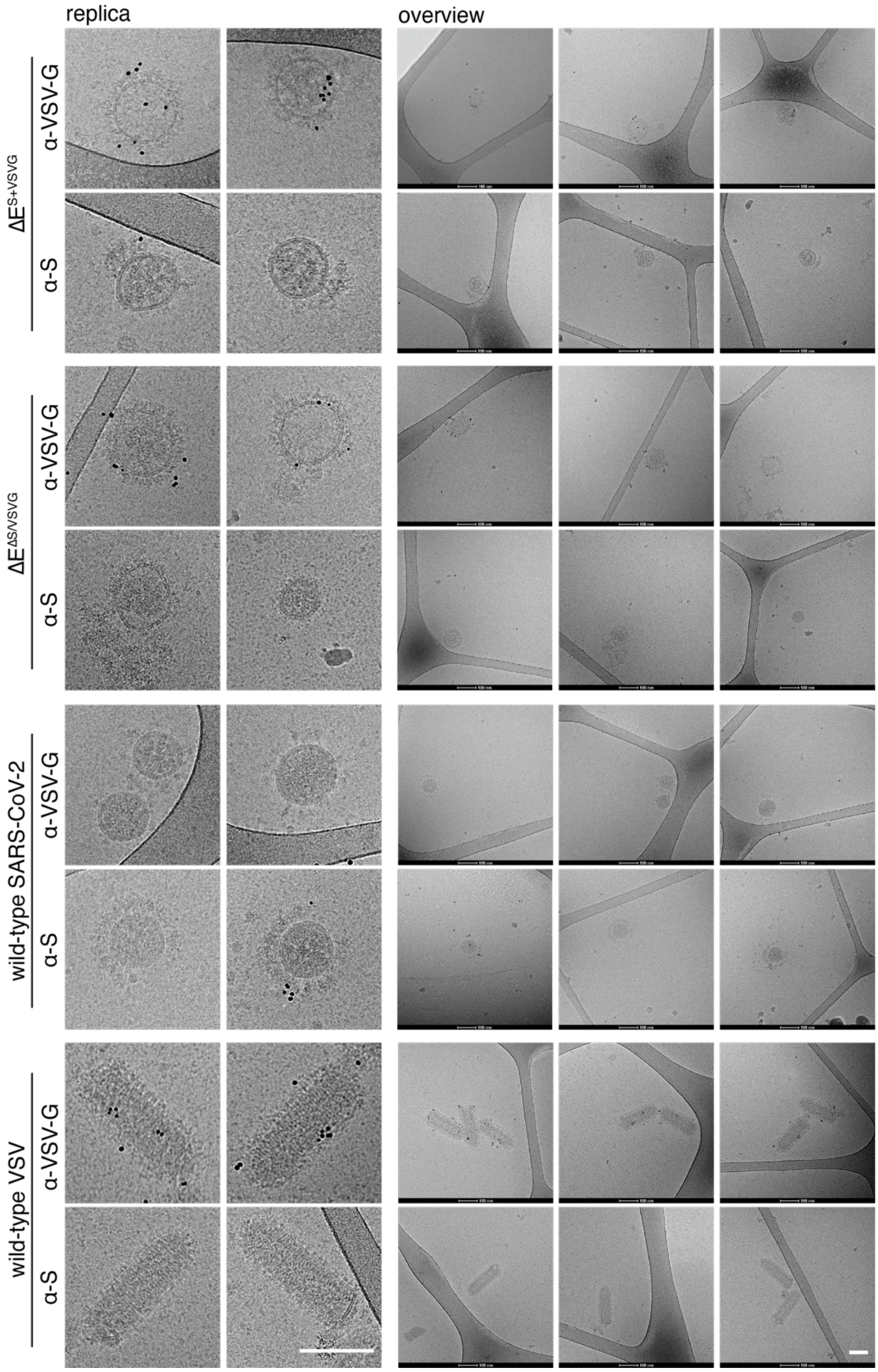

Figure S4. Immunogold-cryo-EM of chimeric particles, related to Figure 2

Supplementary data for immunogold labeling of ΔE^{S+VSVG}, $\Delta E^{\Delta S/VSVG}$, SARS-CoV-2 wild-type, and VSV Indiana wild-type with α-S and α-VSV-G, detected by cryo-EM. Additional replicas corresponding to **Figure 2A** and overview images are shown. Note that α-S has not been detected on all wild-type or ΔE^{S+VSVG} particles. Representative images from *n* = 2 independent experiments are shown.

Scale bar is 100 nm in overview and ROI images.

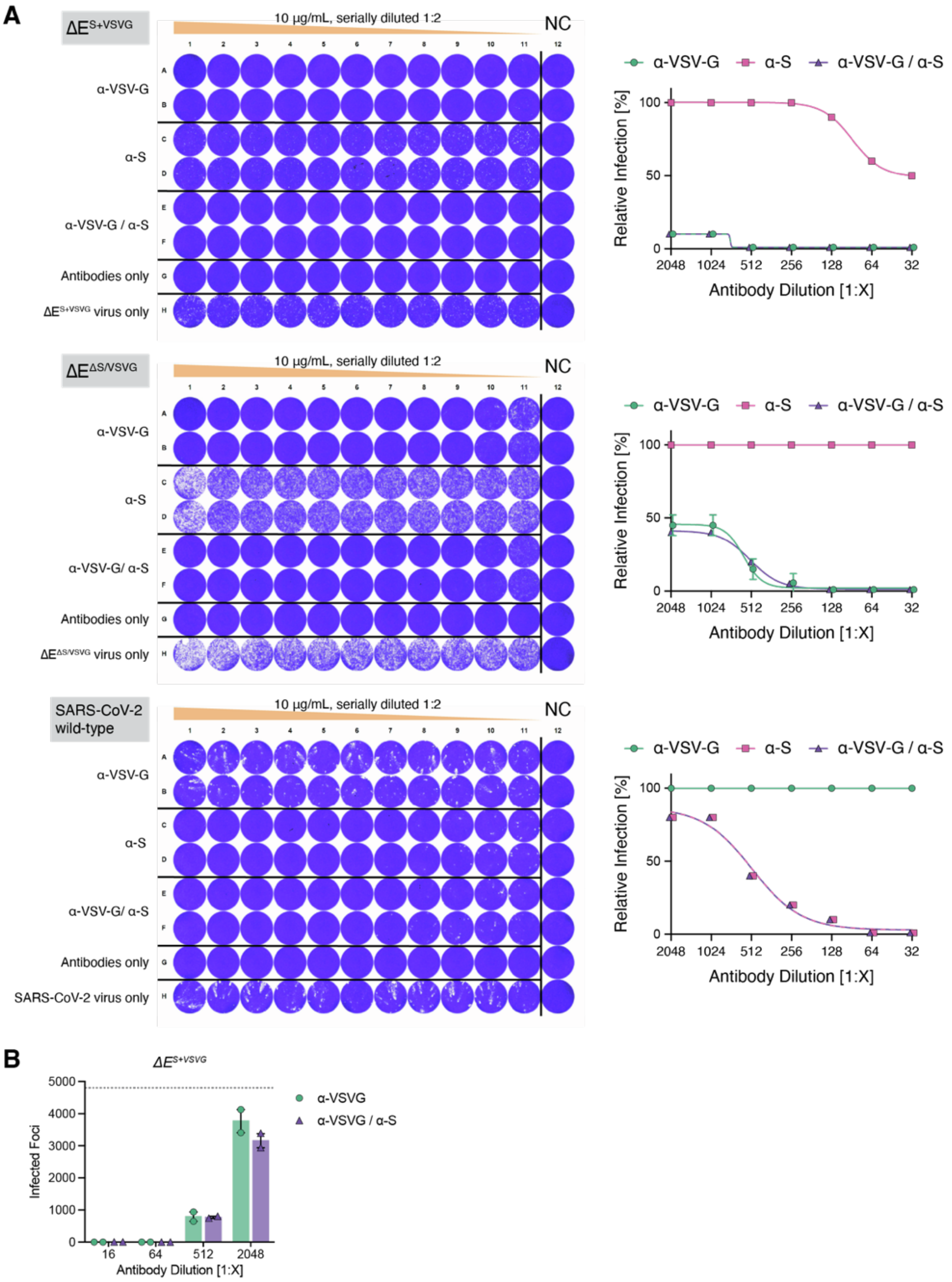

Figure S5. Neutralization of chimeric viruses, related to Figure 2

(**A**) Neutralization assay plates of ΔE^{S+VSVG} (top), $\Delta E^{\Delta S/VSVG}$ (middle), and wild-type SARS-CoV-2 (bottom) incubated with α-VSV-G, α-S, or both (α-VSV-G/ α-S), corresponding to **Figure 2B** (2 technical replicates per condition, mean ± SD plotted). Controls include antibodies or viruses only, and Vero E6 cells only (negative control, NC). Antibody was serially diluted 1:2, starting with 10 μg/mL. Crystal blue-stained plate images were taken with the CTL ImmunoSpot analyzer. Plaque number and size were visually examined and compared to virus-only infections. For wild-type SARS-CoV-2, less virus was used to distinguish individual plaques.

(**B**) Quantification of virus-induced foci with focus forming assay (*n* = 2 technical replicates, mean ± SEM plotted). ΔE^{S+VSVG} virus was mixed with VSV-G or Spike neutralizing antibodies, and infected VeroTMP cells were analyzed for SARS-CoV-2 Nucleocapsid expression.

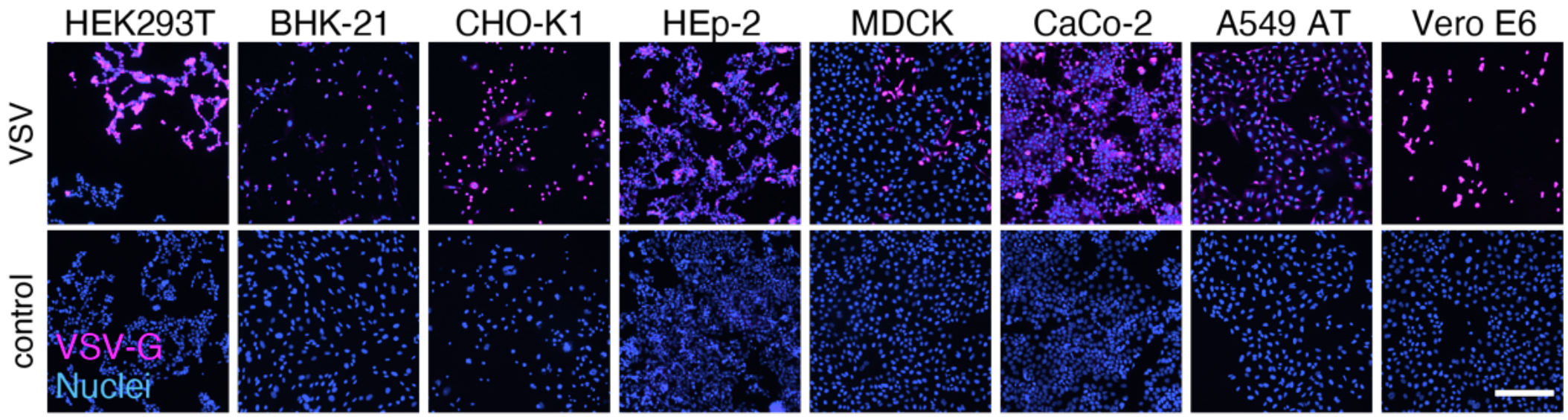

Figure S6. Cell tropism of wild-type VSV, related to Figure 2

HEK293T, BHK-21, CHO-K1, HEp-2, MDCK, CaCo-2, A549 AT, and Vero E6 cells were seeded on coverslips and infected with wild-type VSV or mock control. Cells were fixed 24 hpi and subsequently stained with α-VSV-G to visualize infection events (magenta). Nuclei are in blue. Representative images from *n* = 2 independent experiments are shown.

Scale bar is 200 μm.

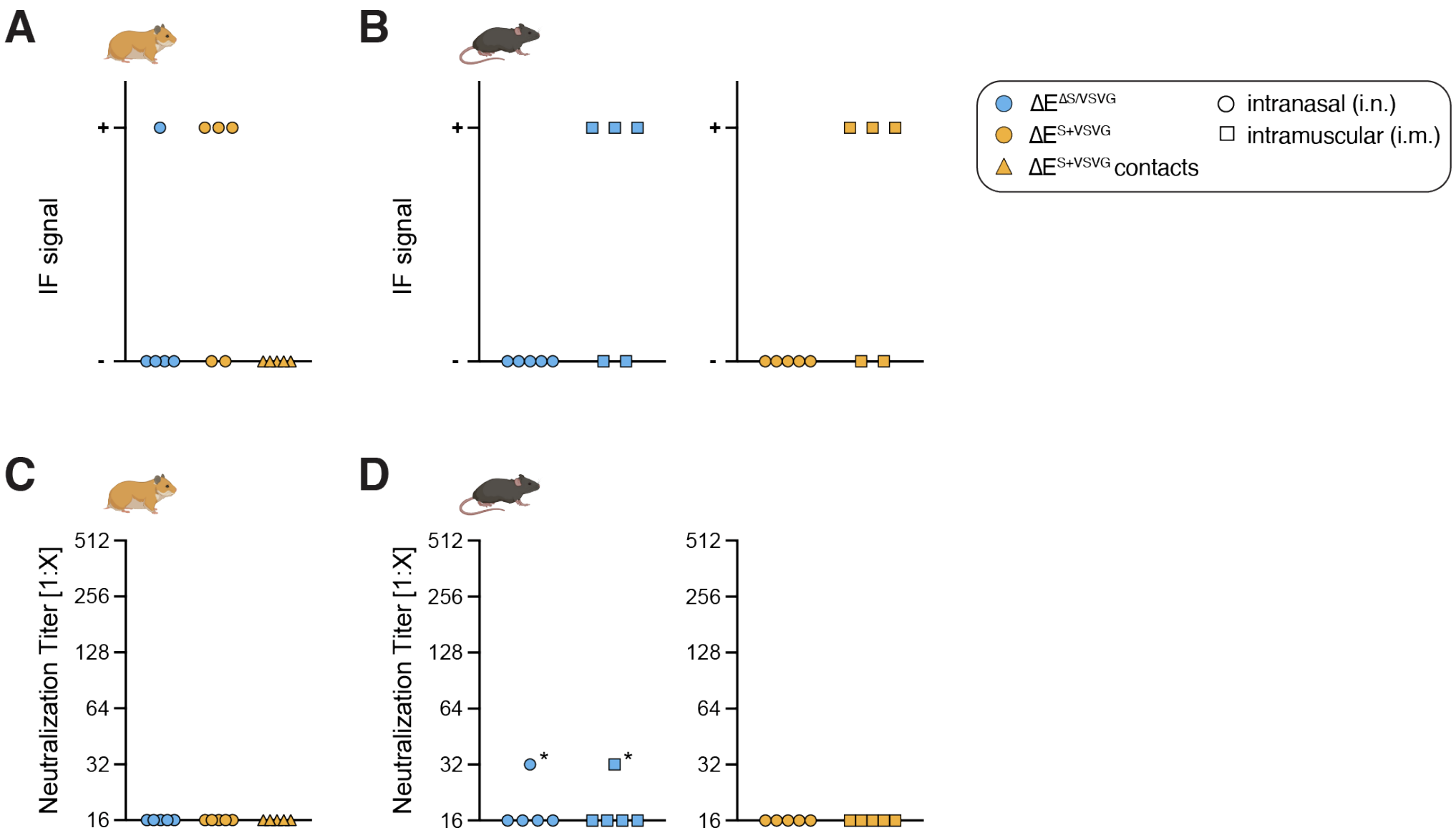

Figure S7. ***In vivo*** **supplement, related to Figures 3 and 4**

(**A**, **B**) Immunofluorescence detection of VSV-G antibodies in sera of inoculated hamsters (**A**) and K18-hACE2 mice (**B**), for both chimeric constructs and application routes. +/- indicates the presence or absence of a fluorescence signal.

(**C**, **D**) Live virus neutralization assay using wild-type SARS-CoV-2 for sera from hamsters (**C**) and mice (**D**). Seronegative threshold at 1:16, or 1:32 for * marked animals, as serum volume was insufficient for a 1:16 starting dilution; these samples were analyzed from 1:32 onward.

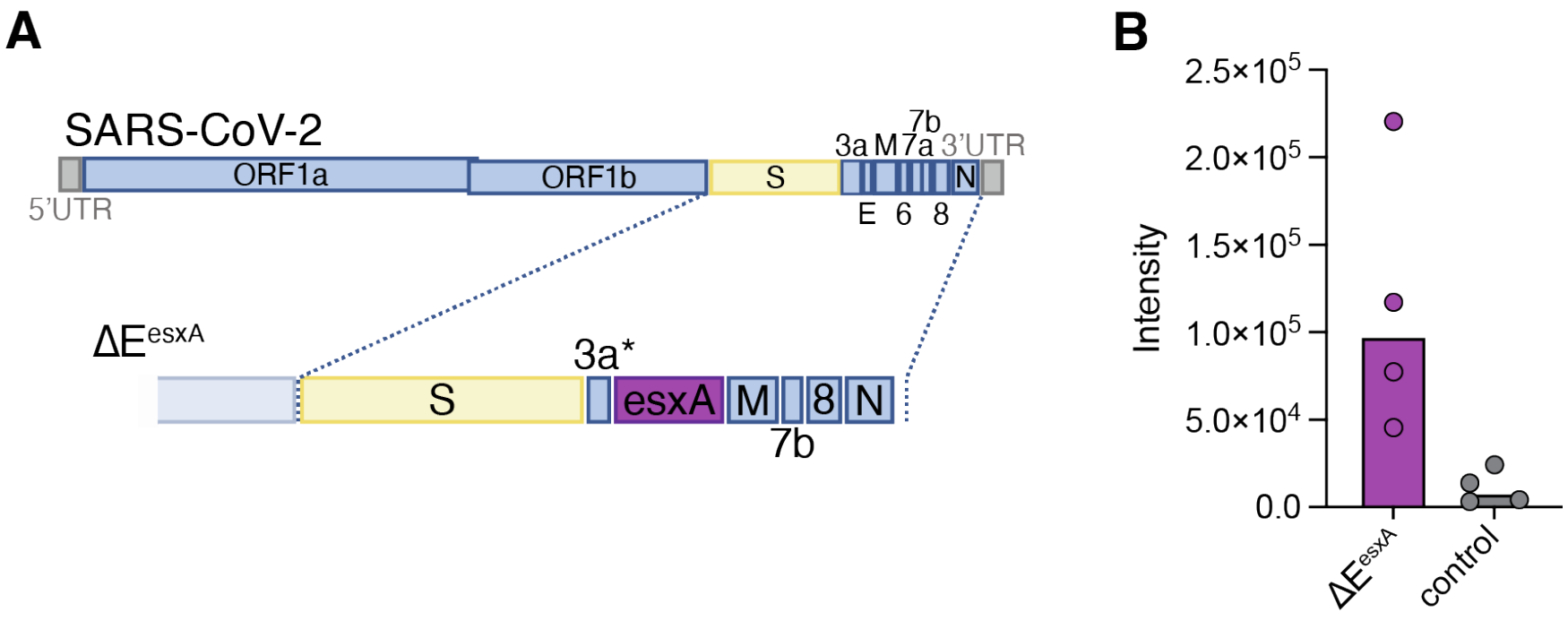

Figure S8. ΔE^{esxA} characterization

(**A**) Genome organization of ΔE^{esxA} with genes indicated. Sequencing data are available in **Table S4**.

(**B**) esxA-protein abundance measured by targeted mass spectrometry, from whole-cell lysates of infected VeroTMP cells or non-infected controls. *n* = 4 technical replicates (individual infected wells), with the geometric mean plotted.
